# Histone demethylase KDM7A regulates bone homeostasis through balancing osteoblast and osteoclast differentiation

**DOI:** 10.1038/s41419-024-06521-z

**Published:** 2024-02-12

**Authors:** Liying Shan, Xiaoli Yang, Xiaoxia Liao, Zheng Yang, Jie Zhou, Xiaoxia Li, Baoli Wang

**Affiliations:** 1 NHC Key Lab of Hormones and Development, Tianjin Key Lab of Metabolic Diseases, Tianjin Medical University Chu Hsien-I Memorial Hospital & Institute of Endocrinology, Tianjin, China; 2 College of Basic Medical Sciences, Tianjin Medical University, Tianjin, China

**Keywords:** Cell signalling, Osteoporosis, Stem-cell differentiation

## Abstract

Histone methylation plays a crucial role in various cellular processes. We previously reported the in vitro function of histone lysine demethylase 7 A (KDM7A) in osteoblast and adipocyte differentiation. The current study was undertaken to investigate the physiological role of KDM7A in bone homeostasis and elucidate the underlying mechanisms. A conditional strategy was employed to delete the Kdm7a gene specifically in osterix-expressing osteoprogenitor cells in mice. The resulting mutant mice exhibited a significant increase in cancellous bone mass, accompanied by an increase in osteoblasts and bone formation, as well as a reduction in osteoclasts, marrow adipocytes and bone resorption. The bone marrow stromal cells (BMSCs) and calvarial pre-osteoblastic cells derived from the mutant mice exhibited enhanced osteogenic differentiation and suppressed adipogenic differentiation. Additionally, osteoclastic precursor cells from the mutant mice exhibited impaired osteoclast differentiation. Co-culturing BMSCs from the mutant mice with wild-type osteoclast precursor cells resulted in the inhibition of osteoclast differentiation. Mechanistic investigation revealed that KDM7A was able to upregulate the expression of fibroblast activation protein α (FAP) and receptor activator of nuclear factor κB ligand (RANKL) in BMSCs through removing repressive di-methylation marks of H3K9 and H3K27 from Fap and Rankl promoters. Moreover, recombinant FAP attenuated the dysregulation of osteoblast and adipocyte differentiation in BMSCs from Kdm7a deficient mice. Finally, Kdm7a deficiency prevented ovariectomy-induced bone loss in mice. This study establish the role of KDM7A in bone homeostasis through its epigenetic regulation of osteoblast and osteoclast differentiation. Consequently, inhibiting KDM7A may prove beneficial in ameliorating osteoporosis.

**Figure:**
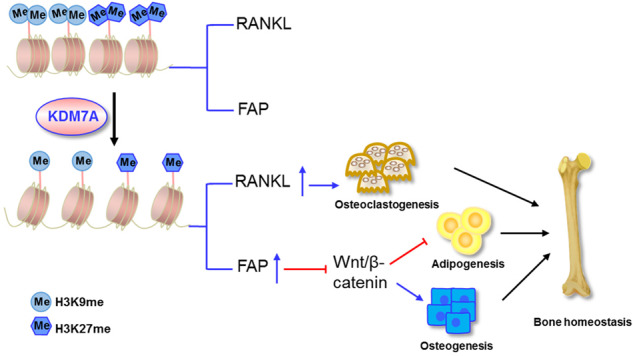
KDM7A suppresses osteoblast differentiation and bone formation through. upregulating FAP expression and inactivating canonical Wnt signaling, and conversely promotes osteoclast differentiation and bone resorption through upregulating RANKL expression. These are based on its epigenetic removal of the repressive H3K9me2 and H3K27me2 marks from Fap and Rankl promoters. As a result, the expression of KDM7A in osteoprogenitor cells tends to negatively modulate bone mass.

KDM7A suppresses osteoblast differentiation and bone formation through. upregulating FAP expression and inactivating canonical Wnt signaling, and conversely promotes osteoclast differentiation and bone resorption through upregulating RANKL expression. These are based on its epigenetic removal of the repressive H3K9me2 and H3K27me2 marks from Fap and Rankl promoters. As a result, the expression of KDM7A in osteoprogenitor cells tends to negatively modulate bone mass.

## Introduction

Bone homeostasis is a complex process fine-tuned by the dynamic well-balanced bone resorption and bone formation [1, 2]. The balance between the activity of osteoclasts, which remove old bone, and that of osteoblasts, which form new bone, determines the health and homeostasis of bone [1, 2]. If one of these two aspects is outpaced by the other, pathological conditions such as osteopetrosis or osteoporosis may occur [2, 3].

Multinucleated osteoclasts arise from osteoclast precursor cells, i.e., haematopoietic stem cell (HSC)-derived monocyte/macrophage lineage cells [4]. The essential factors that control osteoclast differentiation include macrophage/monocyte colony-stimulating factor (M-CSF) and receptor activator of nuclear factor-κB ligand (RANKL), both of which are expressed in bone microenvironment by bone marrow stromal cells (BMSCs), osteoblasts and osteocytes [4, 5]. In contrast, osteoblasts arise from bone marrow-derived mesenchymal stem cells, which also give rise to adipocytes, chondrocytes and myoblasts under appropriate conditions [6, 7]. The differentiation directions toward different lineages, especially those between osteoblasts and adipocytes, compete [8, 9]. Osteoblast differentiation necessitates the presence of two crucial transcription factors, namely runt related transcription factor 2 (Runx2) and osterix (Osx) [10, 11], while adipocyte formation requires peroxisome proliferator-activated receptor γ (PPARγ) and CCAAT element binding protein α (C/EBPα) [9, 12]. Additionally, several signaling pathways are implicated in both osteogenesis and adipogenesis, with Wnt/β-catenin signaling [13] and transforming growth factor-β (TGFβ)/bone morphogenetic protein (BMP) signaling [14] being the most extensively studied.

Post-translational modifications of histone tails, particularly methylation of lysines, play a role in regulating chromatin dynamics and transcription. The steady-state level of histone methylation is controlled by the balance between histone methyltransferases and histone demethylases. Several histone demethylases, such as lysine-specific demethylase 1 (LSD1), lysine demethylase 4B (KDM4B) and lysine demethylase 6B (KDM6B), have been shown to regulate bone cell differentiation [15–19].

Lysine demethylase 7A (KDM7A), which catalyzes the removal of di-methylation marks H3K9m2 and H3K27m2, belongs to the family of Jumonji C (JmjC) domain-containing histone demethylases [20]. KDM7A plays a role in various biological processes, including neural differentiation [21], anterior-posterior development [22], and cancer growth [23, 24]. We have recently reported that KDM7A reciprocally regulates in vitro differentiation of mesenchymal progenitor cells toward adipocytes and osteoblasts [25]. In the current study, we have investigated the physiological role of KDM7A in regulating bone cell differentiation and homeostasis in mice and elucidated the underlying mechanisms.

## Results

### Deletion of Kdm7a in osterix-expressing osteoprogenitor cells promoted bone mass accrual in mice

The Osx-Cre mice (Biocytogen #110131) used in our study have proven to be effective for genetic targeting in osteoblastic lineage [26]. Unlike the Osx1-GFP::Cre line (Jakson lab #006361), which exhibited malocclusion and mild skeletal defects at a young age [27, 28], the Biocytogen Osx-Cre mice did not develop dental abnormalities and had comparable bone volume to Kdm7a^fx/fx^ mice (Fig. S1A–K).

The targeting strategy for the generation of Kdm7a^fx/fx^ mice and Osx-Cre; Kdm7a^fx/fx^ (Kdm7a cKO) mice is illustrated in Supplemental Fig. S2A. Genotyping PCR confirmed the genotypes of the Kdm7a cKO and control Kdm7a^fx/fx^ mice (Fig. S2B). Kdm7a cKO mice were viable, fertile and had no gross physical or behavioral abnormalities. Compared to control mice, Kdm7a cKO mice did not exhibit abnormalities in body length, weight or bone length (Supplemental Fig. S2C–J).

**Fig. 1:**
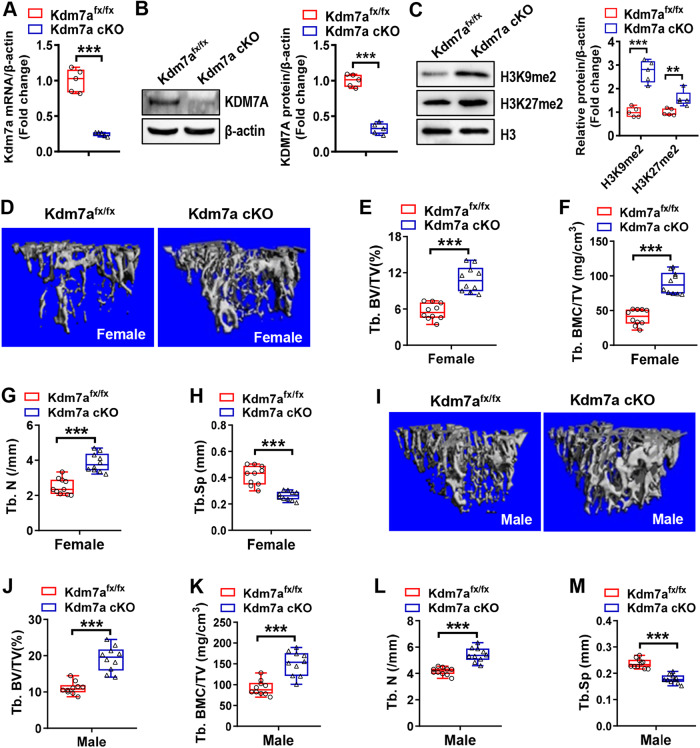
Deletion of Kdm7a in osterix-expressing osteoprogenitor cells promoted bone mass accrual in mice. RT-qPCR and Western blotting were performed to verify the deletion of Kdm7a in long bone BMSCs from Kdm7a cKO mice (**A, B**). The protein levels of H3K9me2 and H3K27me2 were examined (**C**). μCT analyses were performed on the cancellous bone architecture in the proximal tibial metaphysis of 24-week-old female (**D**–**H**) and male (**I**–**M**) mice. The reconstruction images are shown (**D, I**). Histomorphometric parameters of cancellous bone in female (**E**–**H**) and male (**J**–**M**) mice were analyzed. Data are presented as box-and-whiskers plots, *n* = 5 in (**A**–**C**), *n* = 10 in (**D**–**M**). Comparisons were conducted using Student’s *t* test. **p < 0.01, ***p < 0.001.

The conditional deletion of Kdm7a was confirmed in long bone BMSCs by using reverse transcription-quantitative polymerase chain reaction (RT-qPCR) and Western blotting (Fig. 1A, B). Furthermore, deletion of Kdm7a increased H3K9me2 and H3K27me2 levels in BMSCs (Fig. 1C).

We used μCT to assess the changes in bone architecture. In 12-week-old male mice, there was no significant difference in trabecular bone volume/total volume (Tb. BV/TV, %) or trabecular bone mineral content/total volume (Tb. BMC/TV) between the two genotypes. However, the trabecular number (Tb. N, /mm) was slightly increased and trabecular spacing (Tb. Sp, mm) was slightly decreased (Fig. S3). At 24 weeks, however, both male and female cKO mice had greater cancellous bone mass in the proximal metaphysis of tibiae than control Kdm7a^fx/fx^ mice. Specifically, Tb. BV/TV, Tb. BMC/TV, and Tb. N were significantly increased by 96%, 122 and 55%, respectively, while Tb. Sp was reduced by 37% in the female cKO mice (Fig. 1D–H). Additionally, Tb. BV/TV, Tb. BMC/TV, Tb. N and Tb. Sp were changed by similar degrees in male Kdm7a cKO mice as compared to controls (Fig. 1I–M).

Moreover, Tb. BV/TV, Tb. BMC/TV and Tb. N were increased while Tb. Sp was decreased in lumbar vertebrae in Kdm7a cKO mice compared to control mice (Supplemental Fig. S4).

### Osteoblasts were increased and bone formation was enhanced in Kdm7a cKO mice

**Fig. 2:**
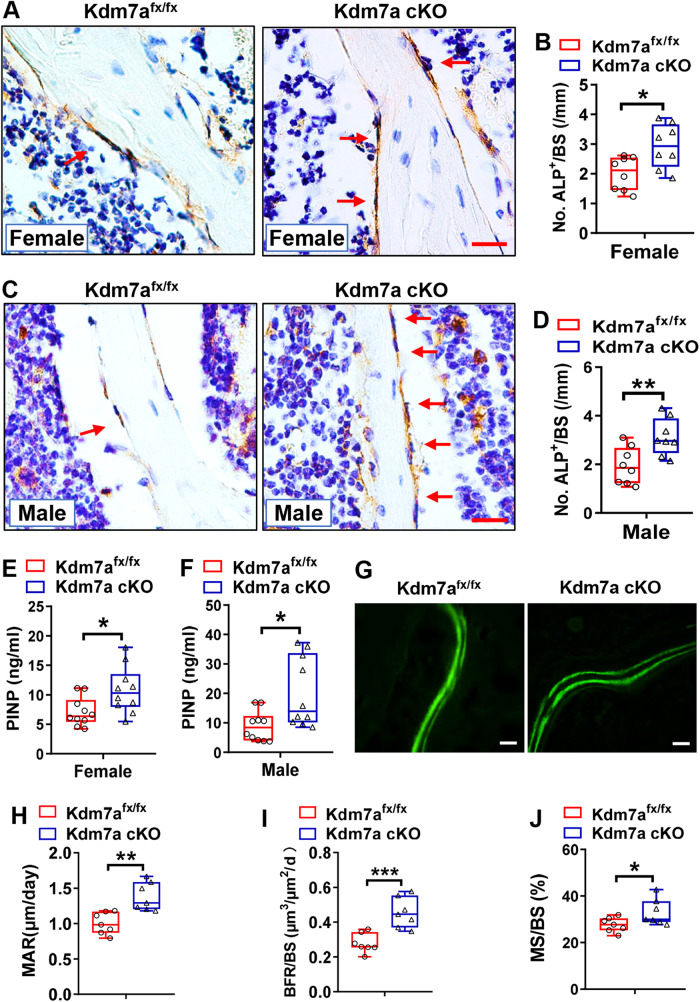
Osteoblasts were increased and bone formation was enhanced in Kdm7a cKO mice. ALP IHC staining was performed on the sections from 24-week-old female (**A, B**) and male (**C, D**) mice (arrows indicate osteoblasts). Image scale in (**A, C**): 20 μm. The number of osteoblasts was counted. Serum PINP levels were measured in female and male mice using ELISA (**E, F**). Calcein double labelling was performed. MAR, MS/BS and BFR/BS were measured and calculated for non-decalcified sections from 24-week-old female mice (**G–J**). Image scale in (**G**): 20 μm. Data are presented as box-and-whiskers plots, *n* = 8 in (**B**, **D**), *n* = 10 in (**E**, **F**), *n* = 7 in (**H**–**J**). Student’s *t* test was performed for statistical analyses. *p < 0.05, **p < 0.01, ***p < 0.001.

We then explored the cellular biological basis underlying the bone phenotypes. There were more alkaline phosphatase (ALP)-positive osteoblasts were more in both female (Fig. 2A, B) and male (Fig. 2C, D) Kdm7a cKO mice than in control Kdm7a^fx/fx^ mice (increased by 46% in females and increased by 62% in males). Consistently, the serum level of procollagen type I N-terminal peptide (PINP), a bone formation marker, was increased in both female and male Kdm7a cKO mice (Fig. 2E, F). Dynamic bone histomorphometric parameters were measured in female mice to further assess bone formation. The results showed that mineral apposition rate (MAR), mineralizing surface/bone surface (MS/BS) and bone formation rate/bone surface (BFR/BS) were greater in Kdm7a cKO mice than in control Kdm7a^fx/fx^ mice (Fig. 2G–J).

### Marrow adipocytes and osteoclasts were reduced and bone resorption was repressed in Kdm7a cKO mice

**Fig. 3:**
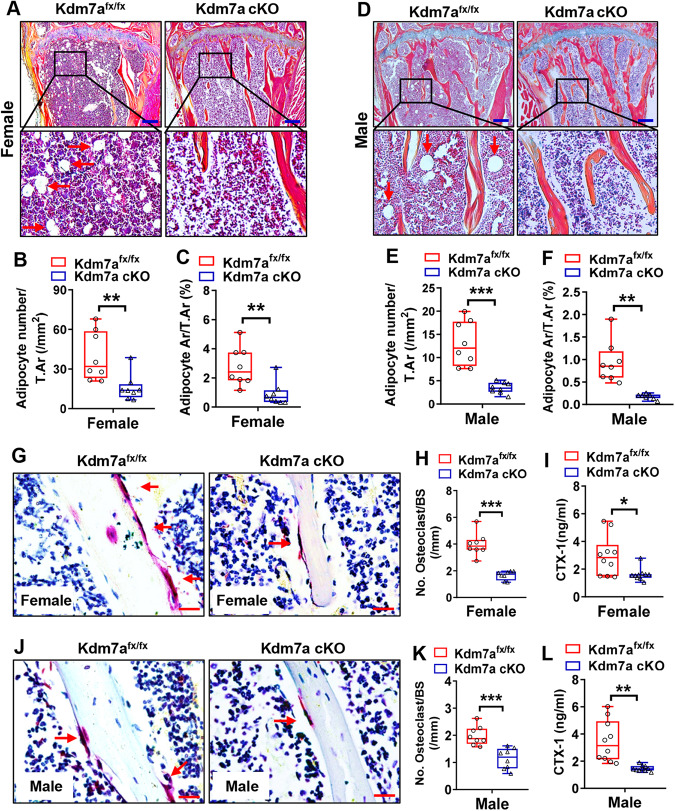
Marrow adipocytes and osteoclasts were reduced and bone resorption was repressed in Kdm7a cKO mice. ABH/OG staining was performed on tibial sections from 24-week-old female (**A**–**C**) and male (**D**–**F**) mice (arrows indicate adipocytes). Image scale in (**A**, **D**): 200 μm. The number and area of adipocytes were quantified. TRAP staining was performed on the tibial sections from 24-week-old female (**G**, **H**) and male (**J, K**) mice (arrows indicate osteoclasts). Image scale in (**G**, **J**): 20 μm. The number of osteoclasts was counted. Serum CTX-1 levels were measured in 24-week-old female (**I**) and male (**L**) mice using ELISA. Data are presented as box-and-whiskers plots, *n* = 8 in (**B, C, E, F, H, K**); *n* = 10 in (**I**, **L**). Student’s *t* test was performed for statistical analyses. *p < 0.05, **p < 0.01, ***p < 0.001.

Alcian blue/hematoxylin/orange G (ABH/OG) staining of the tibial samples showed that the number and area of adipocytes were reduced by 60 and 68%, respectively, in females and by 73 and 81%, respectively, in males of Kdm7a cKO mice (Fig. 3A–F).

Moreover, tartrate-resistant acid phosphatase (TRAP) staining revealed that the number of osteoclasts was reduced by 58% in female Kdm7a cKO mice (Fig. 3G, H) and by 42% in male (Fig. 3J, K) Kdm7a cKO mice compared to their respective controls. Consistently, the serum level of C-terminal telopeptide of type I collagen (CTX-1), a bone resorption marker, was significantly decreased in both female and male Kdm7a cKO mice (Fig. 3I, L).

### Differentiation was dysregulated in osteoprogenitor cells from Kdm7a cKO mice

**Fig. 4:**
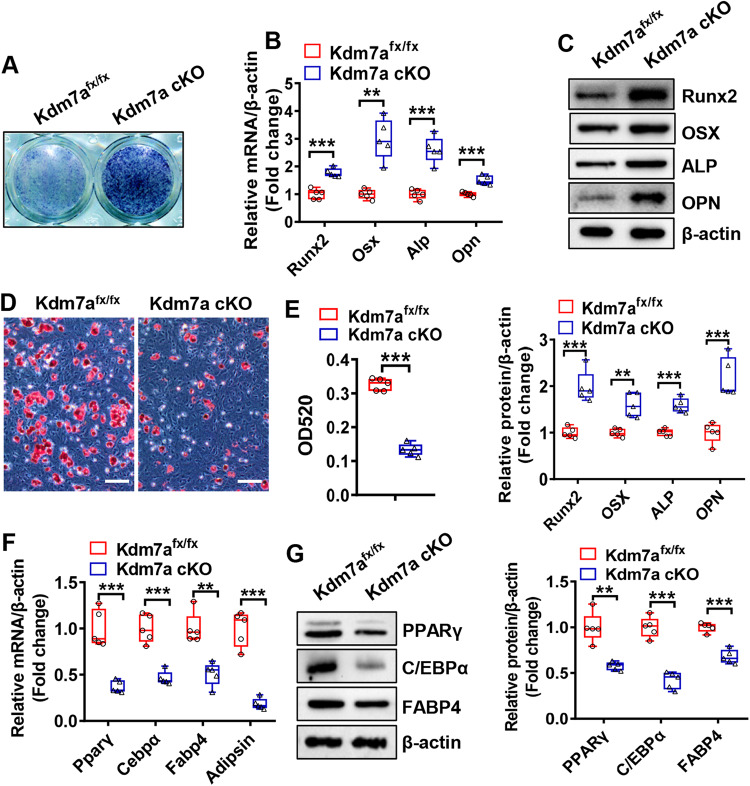
Differentiation was dysregulated in BMSCs from Kdm7a cKO mice. Long bone BMSCs were cultured and induced to allow osteogenic or adipogenic differentiation. ALP staining was performed 14 days after osteogenic treatment (**A**). The mRNA (**B**) and protein (**C**) levels of osteogenic factors were detected 72 h after induction by RT-qPCR and Western blotting, respectively. Oil red O staining was performed 5 days after adipogenic induction, (**D**) and the quantity of dye accumulation in the cells was measured (**E**). The mRNA (**F**) and protein (**G**) levels of adipogenic factors were examined 48 h and 72 h after induction, respectively. Image scale in (**D**): 100 μm. Data are presented as box-and-whiskers plots, *n* = 5. Comparisons were conducted using Student’s *t* test. **p < 0.01, ***p < 0.001.

The potential of long bone BMSCs to differentiate into osteoblasts and adipocytes was assessed. Following osteogenic treatment, osteoblast differentiation from BMSCs was potentiated in Kdm7a cKO mice, as indicated by enhanced alkaline phosphatase (ALP) staining and increased mRNA and protein levels of osteogenic factors such as Runx2, osterix, ALP and osteopontin (Fig. 4A–C). In contrast, under adipogenic condition, adipocyte differentiation from BMSCs was impaired in Kdm7a cKO mice, as indicated by decreased oil red O staining intensity and reduced mRNA and protein levels of adipogenic factors such as PPARγ, C/EBPα, fatty acid binding protein 4 (FABP4) and adipsin (Fig. 4D–G).

Similarly, compared with those of control mice, the differentiation potential of calvarial pre-osteoblastic cells toward osteoblasts was enhanced, whereas that toward adipocytes was suppressed in Kdm7a cKO mice (Fig. S5).

Furthermore, we assessed whether inhibiting KDM7A activity has any impact on the differentiation of wild-type BMSCs. The results showed that treatment with TC-E 5002, a selective inhibitor of the KDM2/7 subfamily, dose-dependently increased the levels of H3K9me2 and H3K27me2, stimulated osteogenic differentiation, and suppressed adipogenic differentiation in BMSCs (Fig. S6A–H).

### Kdm7a deletion in osteoprogenitor cells suppressed osteoclast formation from osteoclast precursor cells

**Fig. 5:**
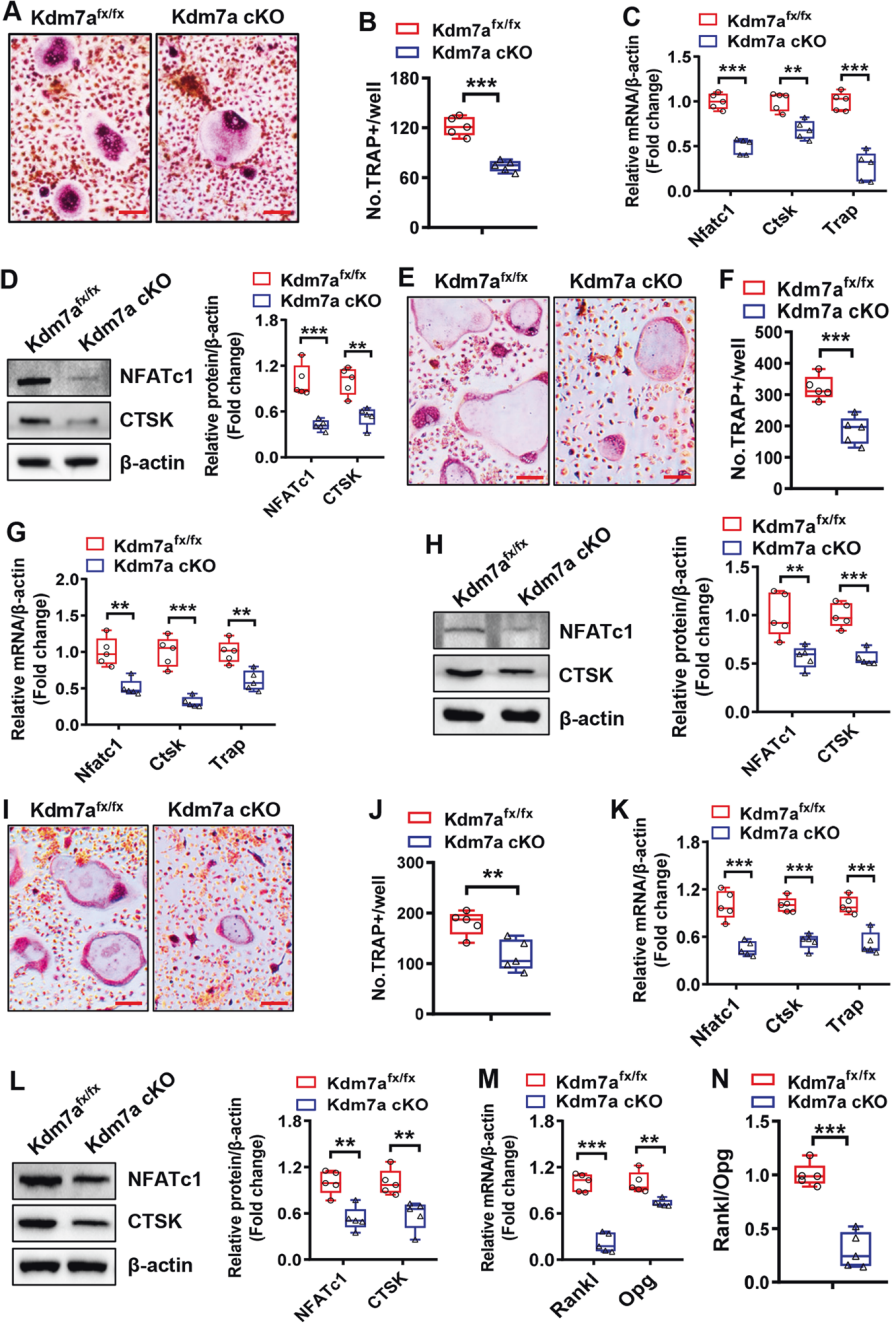
Kdm7a deletion in osteoprogenitor cells suppressed osteoclast formation from osteoclast precursor cells. Bone marrow cells were isolated from 6-week-old Kdm7a cKO and Kdm7a^fx/fx^ mice and induced to allow osteoclast differentiation. Eight days after induction, TRAP staining was performed (**A**) and the number of osteoclasts was counted (**B**). The mRNA (**C**) and protein (**D**) expression levels of osteoclast-specific genes were measured 5 days after induction using RT-qPCR and Western blotting, respectively. Wild-type BMM cells were co-cultured with BMSCs derived from Kdm7a cKO or Kdm7a^fx/fx^ mice in cell-cell contact manner (**E**–**H**) or in transwell manner (**I**–**L**). The co-cultures were induced to allow osteoclast differentiation. Eight days after induction, TRAP staining was performed (**E**, **I**) and osteoclast number (**F**, **J**) was counted. The mRNA (**G**, **K**) and protein (**H**, **L**) expression levels of osteoclast-specific genes were measured 5 days after induction using RT-qPCR and Western blotting, respectively. The mRNA levels of Rankl and Opg in BMSCs were examined (**M**) and the Rankl/Opg ratio was calculated (**N**). Image scale in (**A, E, I**): 100 μm. Data are presented as box-and-whiskers plots, *n* = 5. Comparisons were conducted using Student’s *t* test. **p < 0.01, ***p < 0.001.

Under osteoclastogenic induction, bone marrow cells derived from Kdm7a cKO mice formed fewer osteoclasts and had lower mRNA and protein levels of osteoclastogenic factors such as nuclear factor of activated T cells 1 (NFATc1), cathepsin K (CTSK) and TRAP than those from control Kdm7a^fx/fx^ mice (Fig. 5A–D). We then conducted co-culture experiments to further confirm that KDM7A expression in osteoprogenitor cells regulates osteoclast differentiation. When co-cultured with wild-type bone marrow macrophages (BMM), either through direct cell-cell contact assay (Fig. 5E–H) or transwell assay (Fig. 5I–L), the BMSCs derived from Kdm7a cKO mice exhibited a suppressive effect on osteoclast formation. Additionally, compared with those derived from Kdm7a^fx/fx^ mice, these BMSCs downregulated the mRNA and protein expression levels of osteoclastogenic factors. Of note, the BMSCs derived from Kdm7a cKO mice had lower Rankl mRNA expression and a lower Rankl/osteoprotegerin (Opg) ratio than those from Kdm7a^fx/fx^ mice (Fig. 5M, N).

### Kdm7a deletion in osteoprogenitor cells downregulated fibroblast activation protein α (FAP) and RANKL expression

RNA-seq data revealed differentially expressed genes in the calvarial pre-osteoblastic cells derived from Kdm7a cKO mice versus those from control Kdm7a^fx/fx^ mice, of which 212 genes were upregulated and 329 genes, including Fap and Rankl, were downregulated (Supplemental Fig. S7A, B). KEGG enrichment analysis revealed that these differentially expressed genes are associated mainly with extracellular matrix-receptor interaction, cytokine-cytokine receptor interaction, and PI3K-Akt signaling pathway (Supplemental Fig. S7C).

**Fig. 6:**
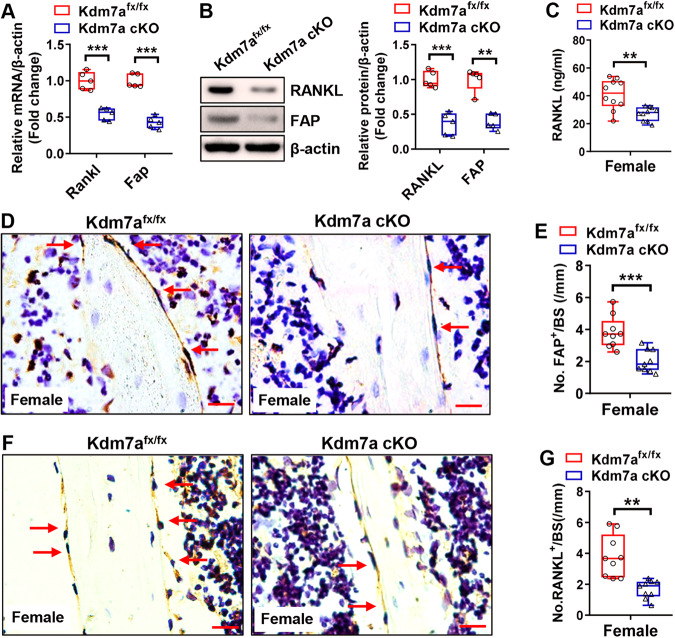
Kdm7a deletion in osteoprogenitor cells downregulated FAP and RANKL expression in female mice. Long bone BMSCs were isolated from Kdm7a cKO and Kdm7a^fx/fx^ mice. The mRNA and protein levels of RANKL and FAP were examined by RT-qPCR and Western blotting, respectively (**A, B**). Serum RANKL levels were measured in 24-week-old female mice using ELISA (**C**). IHC staining of FAP (**D**) and RANKL (**F**) was performed. The numbers of FAP-positive (**E**) and RANKL-positive (**G**) cells on trabeculae were counted. Data are presented as box-and-whiskers plots, *n* = 5 in (**A, B**); *n* = 10 in (**C**); *n* = 9 in (**E, G**). Comparisons were conducted using Student’s *t* test. **p < 0.01, ***p < 0.001.

The downregulation of FAP and RANKL at the mRNA and protein levels, respectively, was verified in Kdm7a-deficient long bone BMSCs by RT-qPCR and Western blotting (Fig. 6A, B). The serum levels of RANKL were significantly lower in both female (Fig. 6C) and male (Fig. S8A) Kdm7a cKO mice than in control mice. Furthermore, IHC staining of the tibial sections showed that both female and male Kdm7a cKO mice had fewer FAP-positive (female: Fig. 6D, E; male: Fig. S8B, C) and RANKL-positive (female: Fig. 6F, G; male: Fig. S8D, E) cells on the trabeculae than Kdm7a^fx/fx^ mice.

### KDM7A targets FAP and RANKL to regulate osteoblast and osteoclast differentiation by removing H3K9me2 and H3K27me2

**Fig. 7:**
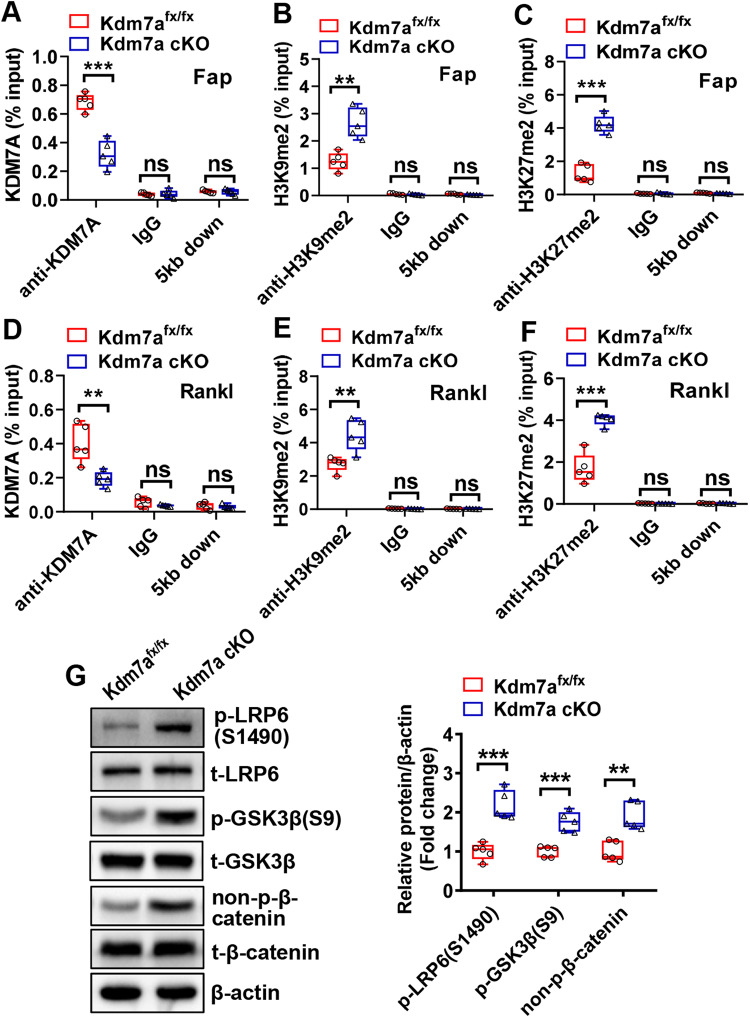
KDM7A regulated Fap and Rankl expression by demethylating H3K9me2 and H3K27me2 on Fap and Rankl promoters. The cell lysates from long bone BMSCs containing chromatin complexes were incubated with anti-KDM7A, anti-H3K9me2, anti-H3K27me2 antibodies or IgG, then the immunocomplexes were captured. ChIP-qPCR was performed to detect the presence of KDM7A, H3K9me2 and H3K27me2 on the promoter of Fap (**A–C**) or Rankl (**D**-**F**). The amplification of a fragment 5 kb downstream of the transcription start site was used as a control. The protein levels of the major components of Wnt/β-catenin pathway were measured in undifferentiated BMSCs using Western blotting (**G**). Data are presented as box-and-whiskers plots, *n* = 5. Comparisons were conducted using Student’s *t* test. **p < 0.01, ***p < 0.001, ns: no significance.

To further elucidate the mechanisms underlying KDM7A function, we performed ChIP assay to evaluate whether KDM7A modulates FAP and RANKL expression through modifying the status of histone methylation at their promoters. The results showed that the deletion of Kdm7a resulted in a reduction in KDM7A binding to the promoters of Fap and Rankl genes in primary BMSCs (Fig. 7A, D). Moreover, the deletion of Kdm7a augmented the presence of H3K9me2 and H3K27me2 on the promoters of Fap and Rankl genes (Fig. 7B, C, E, F). As a control, KDM7A occupancy at the locus 5 kb downstream of the transcription start site was not detected on either Fap or Rankl promoter (Fig. 7A, D), and the deletion of Kdm7a did not affect H3K9me2 or H3K27me2 levels at those loci (Fig. 7B, C, E, F). These data suggest that KDM7A directly regulates Fap and Rankl expression via an epigenetic mechanism.

It was previously reported that FAP inactivates canonical Wnt signaling, we therefore examined if Kdm7a deficiency activates Wnt/β-catenin pathway. Western blotting showed that the protein levels of phospho-low-density lipoprotein receptor-related protein 6 (LRP6) (S1490), phospho-glycogen synthase kinase 3β (GSK3β) (S9) and non-p-β-catenin were increased in Kdm7a-deficient BMSCs (Fig. 7G).

### FAP attenuated Kdm7a deletion-induced dysregulation of osteogenic and adipogenic differentiation

**Fig. 8:**
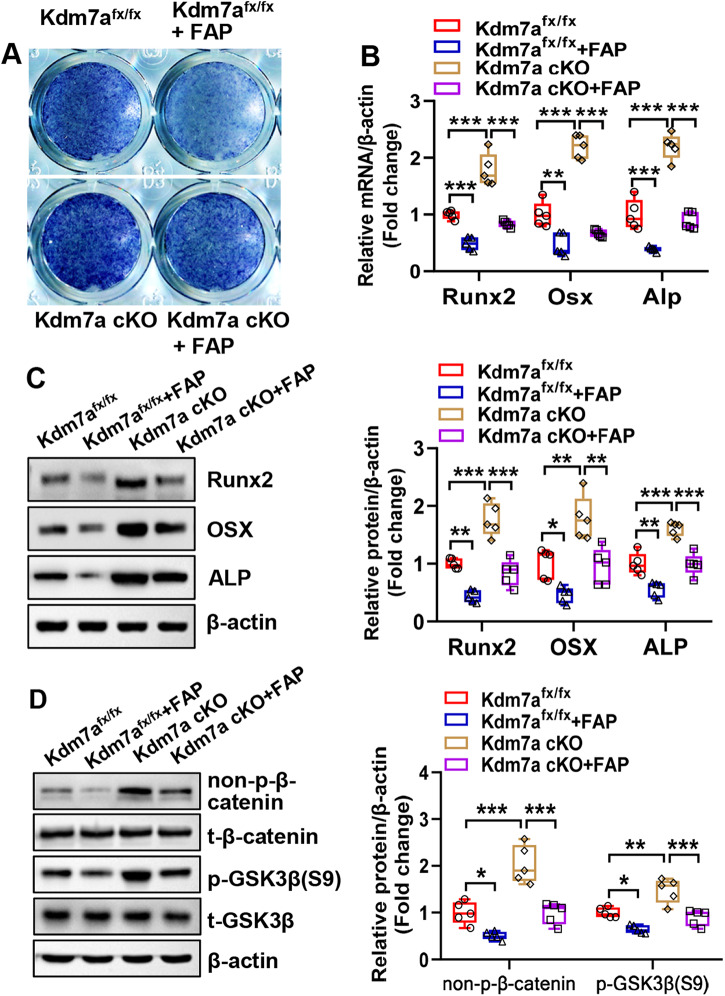
FAP attenuated Kdm7a deletion-induced dysregulation of osteogenic differentiation. Long bone BMSCs were cultured, and treated with 200 ng/ml recombinant FAP followed by osteogenic induction. ALP staining was performed 14 days after osteogenic treatment (**A**). The mRNA (**B**) and protein (**C**) levels of osteogenic factors were examined 72 h after osteogenic treatment. The protein levels of non-p-β-catenin and p-GSK3β(S9) was examined by Western blotting (**D**). Data are presented as box-and-whiskers plots, *n* = 5. Comparisons were conducted using two-way ANOVA followed by Tukey’s test. *p < 0.05, **p < 0.01, ***p < 0.001.

We further investigated whether FAP/Wnt signaling plays a role in mediating the regulation of osteogenesis and adipogenesis by KDM7A. Under osteogenic induction, Kdm7a deletion-induced osteogenic differentiation was attenuated by recombinant FAP. Briefly, the deletion of Kdm7a in BMSCs potentiated ALP staining and increased the mRNA and protein expression levels of osteogenic factors, which was mitigated by the presence of recombinant FAP (Fig. 8A–C). Moreover, Western blotting revealed that Kdm7a deletion-induced activation of Wnt/β-catenin signaling was attenuated by recombinant FAP (Fig. 8D). In contrast, under adipogenic induction, the inhibition of adipogenic differentiation due to Kdm7a deletion was mitigated by recombinant FAP, as revealed by the changes in oil red O staining and expression levels of adipogenic factors (Fig. S9A–D).

### Deletion of Kdm7a in osteoprogenitor cells prevented bone loss in ovariectomized mice

**Fig. 9:**
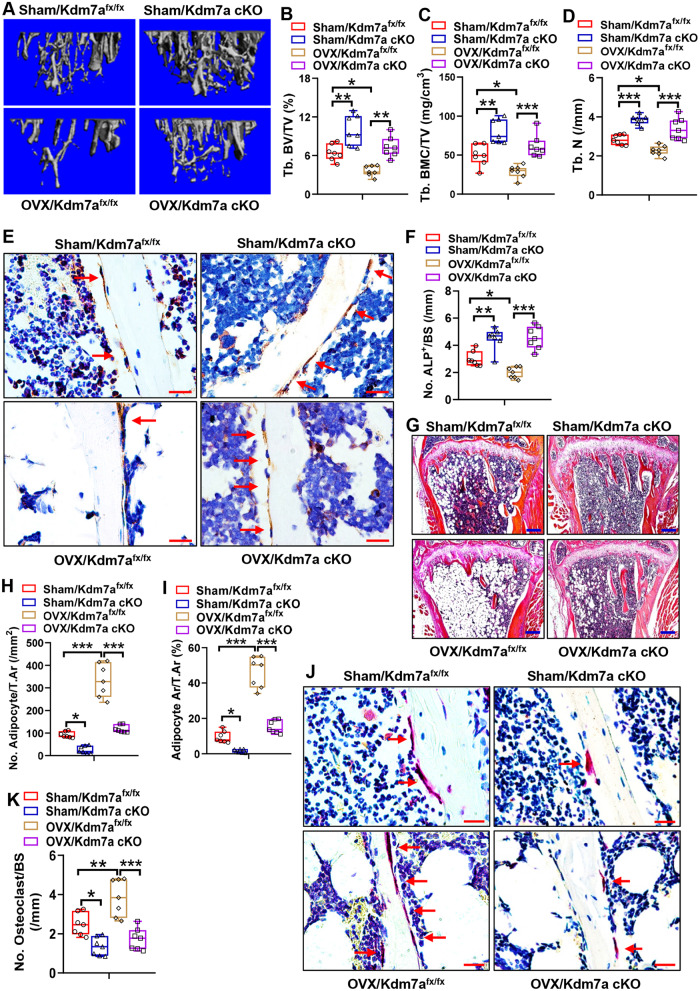
Deletion of Kdm7a in osteoprogenitor cells prevented bone loss in OVX mice. μCT analyses of tibial metaphyseal bone mass were performed and the reconstruction images are shown (**A**). Histomorphometric parameters including Tb. BV/TV, Tb. BMC/TV and Tb. N were measured (**B–D**). ALP IHC staining was performed (arrows indicate osteoblasts) (**E**). Image scale: 20 μm. The number of trabecular osteoblasts in the tibial metaphysis was counted (**F**). H&E staining was performed (**G**). Image scale: 200 μm. The number and area of adipocytes in marrow were quantified (**H, I**). TRAP staining was performed (**J**). Image scale: 20 μm. The number of osteoclast was counted (**K**). Data are presented as box-and-whiskers plots, *n* = 7. Comparisons were conducted using two-way ANOVA followed by Tukey’s test. *p < 0.05, **p < 0.01, ***p < 0.001.

We further investigated whether the deletion of Kdm7a in osteoprogenitor cells has a beneficial role in maintaining bone homeostasis under pathological conditions. Twelve weeks after surgery, μCT analysis revealed a significant bone loss phenotype in OVX/Kdm7a^fx/fx^ mice compared to Sham/Kdm7a^fx/fx^ mice. Specifically, Tb. BV/TV, Tb. BMC/TV and Tb. N were decreased by 44%, 42 and 20%, respectively, in OVX/Kdm7a^fx/fx^ mice (Fig. 9A–D). By contrast, deletion of Kdm7a in osteoprogenitor cells not only increased bone mass in sham-operated mice, but also prevented bone loss in OVX mice. Specifically, compared to those in OVX/Kdm7a^fx/fx^ mice, Tb. BV/TV, Tb. BMC/TV and Tb. N were increased by 102%, 114 and 49%, respectively, in OVX/Kdm7a cKO mice (Fig. 9A–D).

Furthermore, histological analysis revealed that, compared with those in Sham/Kdm7a^fx/fx^ mice, ALP-positive osteoblast number was reduced by 34% (Fig. 9E, F), while marrow adipocyte number, adipocyte area, and osteoclast number were increased by 260%, 386%, and 55% respectively in OVX/Kdm7a^fx/fx^ mice (Fig. 9G–K). In contrast, deletion of Kdm7a in osteoprogenitor cells resulted in an increase in osteoblast number (Fig. 9E, F) and a decrease in marrow adipocyte number, adipocyte area and osteoclast number in both sham and OVX mice (Fig. 9G–K). Specifically, in comparison to those in OVX/Kdm7a^fx/fx^ mice, osteoblast number was increased by 129% (Fig. 9E, F), while adipocyte number, adipocyte area, and osteoclast number were decreased by 65%, 67%, and 55% respectively in OVX/Kdm7a cKO mice (Fig. 9G–K).

## Discussion

We have recently characterized KDM7A as a regulator of osteoblast and adipocyte differentiation in vitro, attributed to its epigenetic regulation of secreted frizzled-related protein 1 (Sfrp1) and C/ebpα gene expression via removing di-methylation marks of histones H3K9 and H3K27 [25]. In the current study, Kdm7a cKO mice in which KDM7A function was lost in osterix-expressing osteoprogenitor cells were generated. Although only mild changes in trabecular number and trabecular spacing were observed in 12-week-old young mice, a dramatic increase in bone mass was observed in 24-week-old female and male Kdm7a cKO mice, indicating an age-related trend. The deletion of Kdm7a resulted in an increase in osteoblast number and a decrease in marrow fat, augmented dynamic bone histomorphometric parameters reflecting osteoblast activity including MAR, MS/BS and BFR/BS, and increased serum levels of PINP, an indicator of bone formation. Consistently, osteogenic differentiation was enhanced in both BMSCs and pre-osteoblastic cells derived from the cKO mice. In contrast, their ability to differentiate into adipocytes was blunted. Moreover, TC-E 5002, a selective inhibitor of the KDM2/7 subfamily, promoted osteogenic differentiation and suppressed adipogenic differentiation. Collectively, these data demonstrate that KDM7A plays a key role in osteoblast differentiation and bone formation in mice.

Interestingly, the Kdm7a cKO mice had fewer osteoclasts and a decreased serum level of CTX-1, an indicator of bone resorption. Moreover, bone marrow cells derived from Kdm7a cKO mice had a decreased tendency toward osteoclast differentiation. Since Kdm7a gene deletion is restricted to osterix-expressing osteoprogenitor cells, it is hypothesized that KDM7A regulation of osteoclasts and bone resorption is based on an indirect mechanism, i.e., KDM7A in osteoprogenitor cells may control osteoclast differentiation via modulating some osteoclast regulator(s) in osteoprogenitor cells. In line with this hypothesis, the Kdm7a deficient BMSCs exhibited an inhibitory effect on osteoclast differentiation when co-cultured with BMM cells in both cell-cell contact and transwell assays. These findings suggest that at least a paracrine regulation is involved in KDM7A-regulated osteoclastogenesis. Therefore KDM7A has dual roles in regulating both osteoblastic bone formation and osteoclastic bone resorption. Notably, deletion of Kdm7a in osteoprogenitor cells led to an increase in osteoblasts, a decrease in adipocytes and osteoclasts, and prevented bone loss in ovariectomized mice. These findings suggest that inhibiting KDM7A in osteoprogenitor cells might be a promising strategy for ameliorating osteoporosis.

By now the mechanism by which KDM7A regulates osteoblast and osteoclast differentiation is the main topic of interest. Transcriptomics analysis revealed hundreds of target genes that were either downregulated or upregulated in Kdm7a deficient cells, among which the most notable two were Fap and Rankl. Of note, the expression of Sfrp1 and C/ebpα was not drastically altered in Kdm7a deficient pre-osteoblastic cells. This suggest that SFRP1 and C/EBPα are not the major players in mediating the physiological role of KDM7A in the bones of mice.

While RANKL is a crucial osteoclastogenic cytokine in both membrane-bound and soluble forms, FAP has recently been identified to be a regulator of osteogenesis and bone formation [29], as well as a pathological factor in osteoarthritis [30]. As a homodimeric integral membrane gelatinase, FAP belongs to the serine protease family and is involved in a variety of biological processes [31]. In bone, genetic loss of Fap gene ameliorates limb trabecular bone loss during aging. Inhibition of FAP activity stimulates bone formation via Wnt/β-catenin signaling [29]. In this study, consistent with the RNA-seq results, we further revealed the downregulation of FAP and RANKL in the BMSCs, calvarial pre-osteoblastic cells and bones of conditional Kdm7a null mice. These highlight the association of altered expression levels of FAP and RANKL with the bone phenotypes observed in Kdm7a cKO mice.

Although there is convincing evidence that bone formation was enhanced in Fap^-/-^ mice and that pharmacological inhibition of FAP promoted osteoblast differentiation [29], the direct effect of FAP on osteoblast differentiation remains to be explored. In this study, recombinant FAP suppressed osteoblast differentiation from control BMSCs. Moreover, Kdm7a deficiency-induced osteoblast differentiation in BMSCs was mitigated by recombinant FAP.

Notably, we observed that recombinant FAP strongly stimulated adipocyte differentiation from BMSCs. Moreover, the Kdm7a deficiency-induced suppression of adipocyte differentiation in BMSCs was mitigated by recombinant FAP. This contradicts the findings of Blomberg et al., who reported the enhancement of adipogenic differentiation in mammary adipose stromal cells derived from Fap^-/-^ mice [32]. This discrepancy may be due to the differences in cell types and/or culturing conditions. It is noted that in Blomberg et al.’s study, the adipose stromal cells were cultured on cell-derived matrices [32], while in our study we cultured the cells on common plates using a standard protocol. However, the exact mechanism for this discrepancy needs to be further investigated.

H3K9me2/3 and H3K27me2/3 are preferentially associated with transcriptional repression [33, 34]. KDM7 subfamily demethylases catalyze the removal of H3K9me2/1 and H3K27me2/1, creating a more permissive chromatin environment for gene transcription [20, 35–37]. Based on our novel finding that KDM7A in osteoprogenitor cells stimulated the expression of Fap and Rankl, we hypothesized that KDM7A functions in regulating osteoblast and osteoclast differentiation by demethylating H3K9me2 and/or H3K27me2 marks on the promoters of Fap and Rankl genes. The subsequent ChIP experiment demonstrated that the presence of KDM7A on either Fap or Rankl promoter was decreased in Kdm7a-deficient cells, indicating that KDM7A can bind to the promoters of Fap and Rankl. Furthermore, conditional deletion of Kdm7a increased H3K9me2 and H3K27me2 on the promoter of Fap and Rankl. These results indicate that KDM7A plays a key role in bone cell fate decision by removing H3K9me2 and H3K27me2 marks, thus derepressing the expression of Fap and Rankl.

Canonical Wnt signaling is widely accepted as a pivotal pathway that controls skeletal development and homeostasis by regulating the differentiation of BMSCs [13, 38, 39]. Although the exact downstream substrate involved remains to be determined, FAP has been shown to inactivate Wnt/β-catenin signaling [29]. We have previously reported that KDM7A inactivats canonical Wnt pathway in vitro [25]. Herein we observed the upregulation of canonical Wnt signaling activity in Kdm7a-deficient BMSCs, which was mitigated by recombinant FAP. These findings suggest that the downregulation of canonical Wnt signaling by KDM7A is at least partially mediated by FAP.

In conclusion, our work has characterized the physiological role of KDM7A in osteoprogenitor cells as a regulator of bone homeostasis. It suppresses osteoblast differentiation and bone formation through upregulating FAP expression and inactivating canonical Wnt signaling, and conversely promotes osteoclast differentiation and bone resorption through upregulating RANKL expression. These are associated with its epigenetic removal of the repressive H3K9me2 and H3K27me2 marks from Fap and Rankl promoters. As a result, KDM7A in osteoprogenitor cells tends to negatively modulate bone mass, and downregulation of KDM7A ameliorates bone loss in OVX mice. Our study provides new insights into the epigenetic mechanisms underlying bone homeostasis.

One limitation of the study is that the physiological role of KDM7A is concluded mainly based on animal data. Further research needs to be conducted to more conclusively determine the relevance of KDM7A to human bone disorders such as osteoporosis.

## Materials and methods

### Animals

The Osx-cre mouse which targets Osx-expressing osteoprogenitor cells was purchased from Biocytogen (Beijing, China; https://www.biocytogen.com.cn/models/tool/Cre/B-Sp7-iCre-mice.html; #110131). It was generated by placing an F2A-iCre sequence between exon 2 and 3’UTR of osterix gene in C57BL/6 embryonic stem cells. The Kdm7a floxed mouse which contains LoxP sites flanking exon 2 of mouse Kdm7a was generated via CRISPR/Cas9 technology by GemPharmatech (Nanjing, China). Kdm7a^fx/fx^ mice were crossed with Osx-cre mice to generate the knock-out line Osx-cre; Kdm7a^fx/fx^ (Kdm7a cKO). The sequences of primers used for genotyping are listed in Supplemental Table 1.

To investigate the bone phenotypes of Kdm7a cKO mice, Kdm7a^fx/fx^ mice were used as controls. To investigate whether deletion of Kdm7a in osteoprogenitor cells prevents ovariectomy (OVX)-induced bone loss, 12-week-old female cKO and Kdm7a^fx/fx^ mice were subjected to OVX or sham operation under anesthesia. For each genotype, the mice for OVX and sham operations were randomly allocated. Twelve weeks after surgery, tibial samples were collected and bone morphometric parameters were analyzed via μCT followed by histological staining.

Animal experiments were carried out the Animal Research: Reporting of In Vivo Experiments (ARRIVE) guidelines [40]. The sample size for the animal experiments was determined based on a survey of data from published papers. Five mice were housed per cage in a standard individually ventilated cage (IVC) system (Tecniplast, Italy) in an SPF facility maintained under controlled temperature (22 °C) and humidity (50–60%), as well as a 12 h dark:12 h light cycle. The mice were given free access to autoclaved water and food. All the experimental animals were located in the same animal room and subjected to the same environmental conditions to avoid confounding factors. All animals with correct genotypes were included in data collection. The animal protocol and experimental methods were approved by the Animal Ethics Committee of Tianjin Medical University and Chu Hsien-I Memorial Hospital.

### μCT analysis

Tibial and lumbar vertebral samples from 24-week-old mice were scanned using a Scanco viva CT80 (Scanco Medical AG, Switzerland) operated with the following parameters: voxel size of 10.4 μm, energy at 55 kVp, intensity of 145 μA, FOV/Diameter of 31.9 mm, and 300 msec integration time. The measurements were quantitatively analyzed. Three-dimensional reconstructions were made using Scanco software. The region of interest for histomorphometric parameters was selected in the 1 mm region starting 0.2 mm below the growth plate.

### Cell cultures

Primary BMSCs were isolated from the marrow of 6-week-old mice and cultured following a previously described procedure [41]. Pre-osteoblastic cells were isolated from the calvaria of 3-day-old mice. Briefly, the calvaria were minced into 1 mm×1 mm tissue blocks and subjected to five rounds of digestion using an enzyme mixture consisting of 0.25% trypsin and 0.1% type I collagenase at 37 °C. The cells collected from the last 4 digestions were pooled, centrifuged, resuspended and cultured in α-MEM supplemented with 10% FBS.

The cells were passaged at 80% confluence and those at passages 3–5 were induced to allow osteogenic and adipogenic differentiation following a previously published protocol [41]. In certain experiments aimed at investigating the underlying mechanisms, cells were treated with 200 ng/ml recombinant FAP (Sino Biological), or TC-E 5002 (MedChemExpress, USA), a selective inhibitor of the KDM2/7 subfamily, at doses of 10, 20 and 40 μmol/l.

### Oil red O staining

After 5–6 days of adipogenic differentiation, differentiated adipocytes were fixed with 4% paraformaldehyde for 10 min, washed with PBS, and rinsed with 60% isopropanol. The cells were stained with 0.24% fresh oil red O solution for 5 min. To quantify the intensity of the staining, the oil red O dye retained in the cells was extracted using isopropanol, and the optical density was measured at 520 nm.

### Staining of differentiated osteoblasts

Differentiated osteoblasts were subjected to ALP staining after 14 days of osteogenic induction, or to alizarin red staining after 21 days of induction as per a previously published procedure [41]. To quantify the intensity of the alizarin red staining, the stained cultures were destained with a solution containing 0.5 mol/L HCl and 5% SDS for 10 min, and the absorbance of the solution was read at 415 nm.

### ALP activity measurement

Cell extract was diluted 5-fold and incubated with para-nitrophenyl phosphate (pNPP) liquid substrate (Beyotime Biotech, Shanghai, China) for 5 min at 37 °C. The absorbance was measured at OD405 on a microplate reader after addition of the termination solution. For normalization, total protein concentration was measured using a BCA protein assay kit (EpiZyme, Shanghai, China) and OD562 was measured. The OD405 value was divided by OD562 value to generate a standardized value for each sample.

### RT-qPCR

Total RNA was extracted from cultured cells using a kit (Omega Biotek, Norcross, GA) or from tissues using RNAiso (Vazyme, Nanjing, China). cDNA synthesis and quantitative PCR amplification were performed using PerfectStart Uni RT&qPCR Kit (TransGen, Beijing, China). The expression levels of target genes were calculated by comparative Ct (ΔΔCt) method using β-actin as an internal reference. The primer sequences are listed in Supplemental Table S1.

### RNA-seq

Total RNA was isolated from calvarial cells of Kdm7a cKO mice and Kdm7a^fx/fx^ mice. The RNA-seq transcriptome library was prepared with 1 μg of total RNA, and subsequently sequenced on the MGISEQ-2000 platform (sequencing length: PE150) by Beijing Genomics Institute (Wuhan, China). Gene Ontology functional enrichment and Kyoto Encyclopedia of Genes and Genomes (KEGG) pathway analyses were conducted.

### Western blotting

Cells were lysed in RIPA lysis buffer and protein concentration was measured using a BCA assay kit (EpiZyme). The proteins were separated via 10% SDS-PAGE, and then transferred onto nitrocellulose membranes. The membranes were incubated with primary antibodies at 4 °C overnight, followed by incubation with horseradish peroxidase-conjugated secondary antibodies. Protein bands were detected with a chemiluminescence kit (Epizyme). The primary antibodies used are listed in Supplemental Table S2.

### Chromatin immunoprecipitation (ChIP) assay

ChIP assay was performed using an ABclonal kit (Wuhan, China). Briefly, mouse BMSCs were incubated with 1% formaldehyde for cross-linking, and then sonicated to generate 200–500 bp genomic DNA fragments. The cell lysates containing chromatin complexes were incubated overnight at 4 °C with 5 μg of anti-KDM7A (Biorbyt, orb67004), anti-H3K9me2 (ABclonal, A2359), anti-H3K27me2 antibody (ABclonal, A2362) or IgG, and then incubated with protein A/G beads to capture the immunocomplexes for 2 h at 4 °C. The de-crosslinked and purified DNA was used as a template to PCR-amplify mouse Rankl or fibroblast activation protein α (Fap) promoter sequences. Data are expressed as the percentage of input DNA. The primer sequences for ChIP-qPCR are listed in Supplemental Table S1.

### Osteoclast differentiation from bone marrow cells

Bone marrow cells isolated from the long bones of 6-week-old Kdm7a cKO mice or Kdm7a^fx/fx^ mice were seeded into a 48-well plate at the density of 4×10^5^ cells/well. The cells were cultured in α-MEM supplemented with 10% FBS and 20 ng/ml M-CSF (Sino Biological, Beijing, China) for 3 days, and then induced with 20 ng/ml M-CSF and 5 × 10^−7^ mol/l 1,25(OH)_2_D_3_ (APExBIO, USA) to allow osteoclast differentiation. The medium was refreshed every 3 days. After 8 days of induction, the cultures were stained with a TRAP staining kit (Sigma-Aldrich, USA). TRAP-positive multinucleated osteoclasts (≥3 nuclei) were counted.

### Osteoclast differentiation from co-cultures

Bone marrow cells were collected from 10- to 14-day-old C57BL/6 J mice and cultured in α-MEM containing 10% FBS. After 24 h, the nonadherent BMM cells were collected.

For cell-cell contact co-culturing, BMM cells were seeded into a 24-well plate at the density of 5×10^5^ cells/well. After 3 days of culturing in α-MEM supplemented with 10% FBS and 20 ng/ml M-CSF, the BMSCs from Kdm7a cKO or Kdm7a^fx/fx^ mice were seeded into the cultures at the density of 5,000 cells/well. The co-cultures were induced with 20 ng/ml M-CSF and 5 × 10^−7^ mol/l 1,25(OH)_2_D_3_ to allow osteoclast differentiation.

Transwell co-culturing was conducted using 24-well transwell plates with 0.4 μm membrane pores (Biofil, Guangzhou, China). Briefly, BMM cells were seeded into the transwell bottom compartment at the density of 5×10^5^ cells/well and cultured in the presence of 20 ng/ml M-CSF. The BMSCs from Kdm7a cKO or Kdm7a^fx/fx^ mice were seeded into the top compartment at the density of 5,000 cells/well and cultured in the presence of 5 × 10^−7^ mol/l 1,25(OH)_2_D_3_. The medium was refreshed every 3 days. After 8 days of induction, TRAP staining was performed and osteoclasts were counted.

### Enzyme-linked immunosorbent assay (ELISA)

ELISA was conducted following the manufacturer’s protocols to measure the levels of serum CTX-1 (SAB, College Park, MD, USA), RANKL (SAB), and PINP (SAB).

### Histological staining

Bone samples were fixed with 10% formalin for 3 days, decalcified with 14% EDTA (pH 7.4) for 21 days, and embedded in paraffin. Then the samples were cut into 4-μm thick sections. Deparaffinized and rehydrated sections were stained with standard ABH/OG and hematoxylin/eosin (H&E). Marrow adipocyte number was counted and adipocyte area was measured. To identify osteoclasts, TRAP staining was performed using staining solution containing 0.1 mol/l sodium acetate (pH 5.0), 0.5 mol/l L-(+) tartaric acid, 0.2 mmol/l naphthol AS-MX and 1.5 mmol/l fast red violet LB salt. TRAP-positive osteoclasts were counted. The region of interest is 1 mm long starting from 0.2 mm below the growth plate.

### Immunohistochemical staining

For antigen repair, the deparaffinized and rehydrated sections were digested with 0.075% trypsin at 37 °C for 15 min. After removing endogenous peroxidase with 3% H_2_O_2_ and blocking with 1% BSA, the sections were probed with primary antibodies overnight at 4 °C, and then incubated with HRP-conjugated secondary antibodies for 2 h at 4 °C. The primary antibodies used included anti-ALP (Huabio, Hangzhou, China), anti-RANKL (WL00285, Wanleibio, Shenyang, China) and anti-FAP (WL04890, Wanleibio). A 3,3’-diaminobenzidine (DAB) staining kit (GeneTech, Shanghai, China) was applied to visualize the staining. The nuclei were counterstained with hematoxylin.

### Dynamic bone histomorphometry

Twenty-four-week-old female mice were injected intraperitoneally twice with calcein (Sigma, USA) at the dose of 10 mg/kg 2 and 7 days, respectively, prior to sacrifice. The femur samples were dehydrated in a graded series of ethanol and embedded, non-decalcified, in methyl methacrylate resin and sectioned at 8-μm thickness using a microtome (Leica RM2155, Wetzlar, Germany). The region of interest is 1 mm long starting from 0.2 mm below the growth plate. The unlabelled perimeter, single-labelled perimeter, double-labelled perimeter, and the area between the double labels were measured using the OsteoMeasure software (OsteoMetrics, Atlanta, GA, USA). Histomorphometric parameters, including MAR, MS/BS, and BFR/BS were calculated.

### Data illustration and statistical analysis

Cell-based experiments were performed independently 5 times. The relative levels of mRNAs and proteins in the control group were set to 1. Statistical analyses were performed using GraphPad Prism 8.0 in a blinded manner. Data are presented as box-and-whiskers plots in which the boxes extend from the 25th to 75th percentiles, and the lines in the middle of the boxes indicate medians; all individual values are shown and the whiskers represent the minimum and maximum values.

Data were initially subjected to normal distribution analysis using Kolmogorov-Smirnov nonparametric test. For the comparisons between two groups, two-tailed unpaired independent Student’s *t* test was conducted, with Welch’s correction when there was unequal variance. For comparisons among multiple groups, One-way ANOVA followed by Dunnett’s test or two-way ANOVA followed by Tukey’s test was conducted. *P* < 0.05 was regarded as significantly different.

### Supplementary information

Supplemental figures and tables

aj-checklist

Original WB images

Dataset 1

## Data Availability

The RNA-seq datasets and original images for Western blotting are included in the supplemental information. All other data are available from the corresponding author upon reasonable request.
