# Supplementary material for: Histone demethylase KDM7A regulates bone homeostasis through balancing osteoblast and osteoclast differentiation
Source: Cell Death Dis. 2024 Feb 12;15(2):136. doi: 10.1038/s41419-024-06521-z (PMC10861515; doi:10.1038/s41419-024-06521-z)
Supplement: Supplementary file 1 — Supplemental figures and tables [file 41419_2024_6521_MOESM1_ESM.pdf]

## Supplemental material

## Figure S1

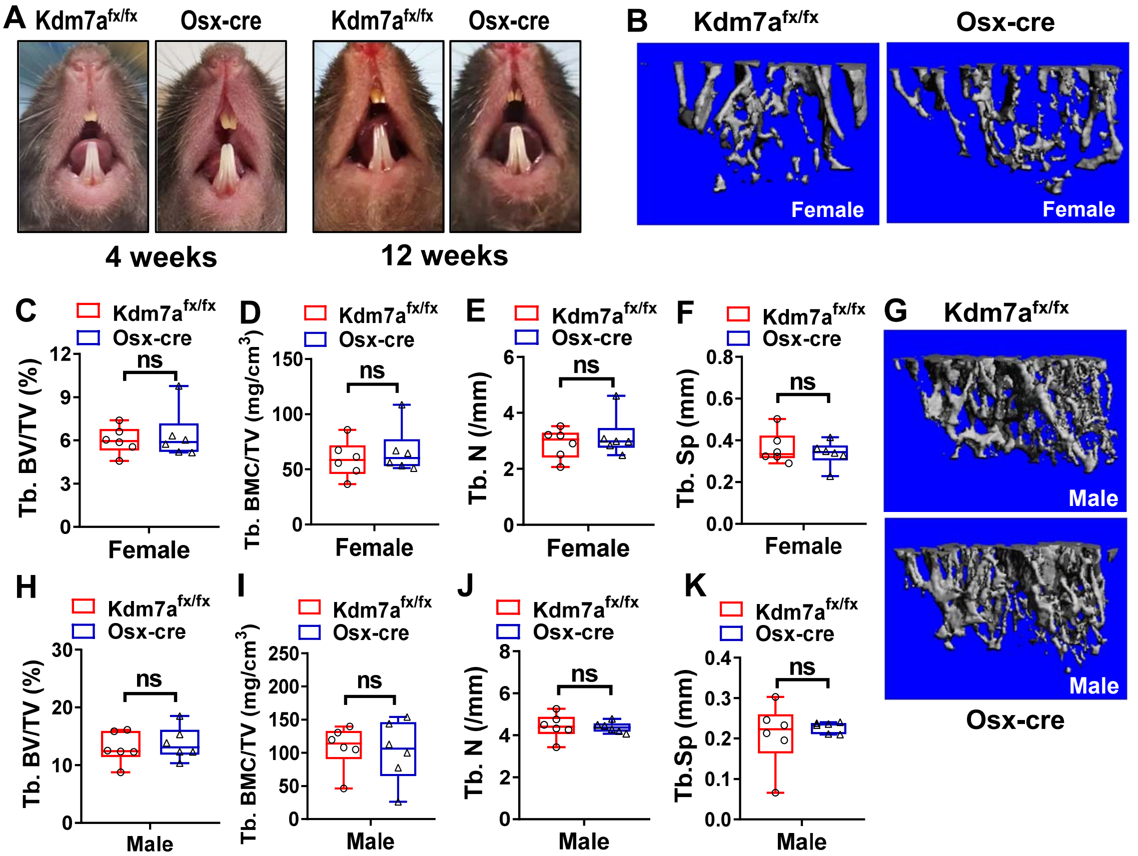


**Figure S1. Osx-Cre mice did not show dental and skeletal defects.** Gross appearance of incisors in 4-week-old and 12-week-old mice was shown (A). μCT analyses were performed on the tibial metaphyseal bone of 24-week-old female (B-F) and male (G-K) mice. The reconstruction images are shown (B, G). Histomorphometric parameters were analyzed in female (C-F) and male (H-K) mice. Data are presented as box-and-whiskers plots, n=6. Student's t test was performed for statistical analyses, ns: no significance.

## Figure S2

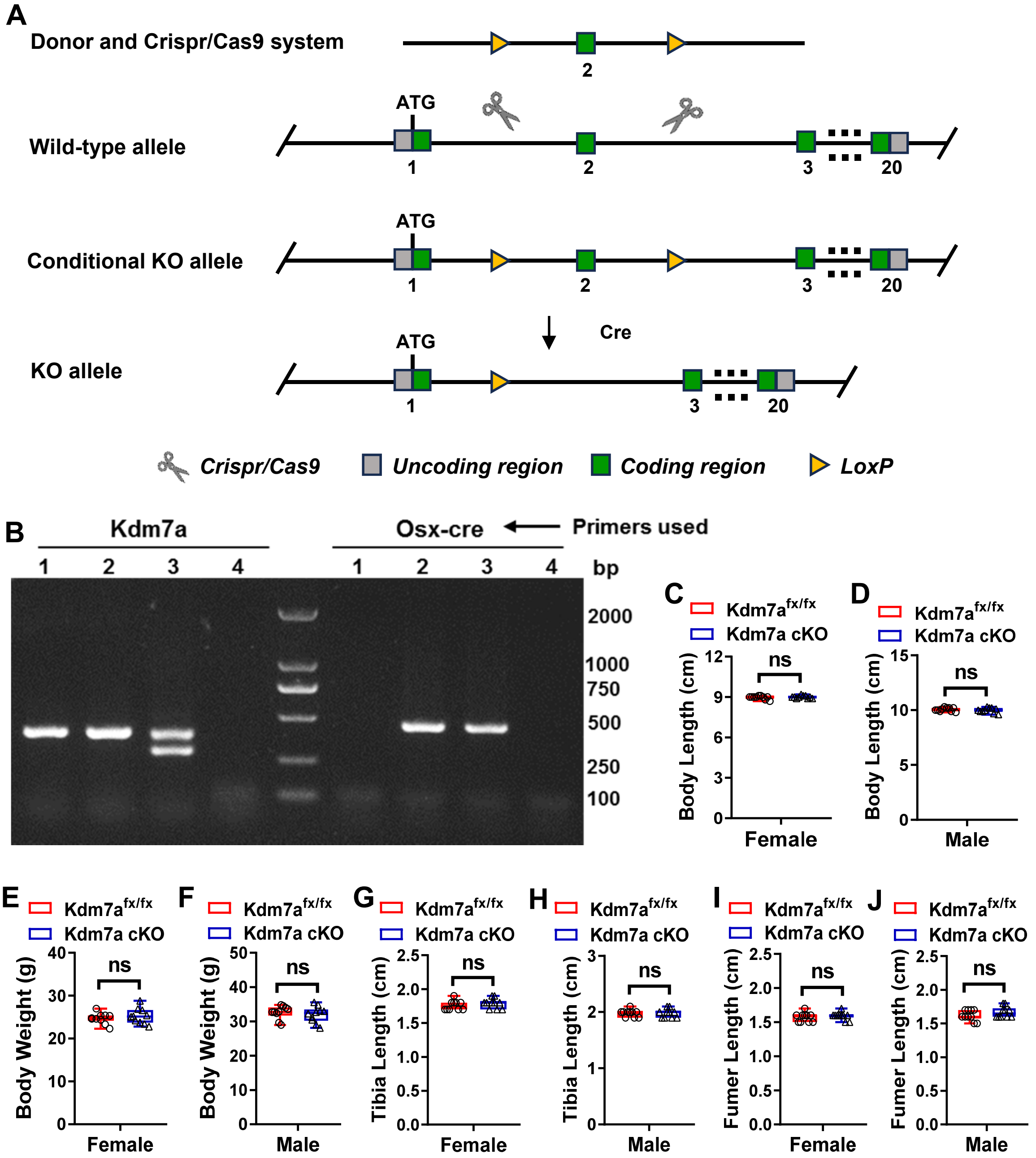


**Figure S2. Kdm7a cKO mice did not show abnormalities in body length, weight and bone length.** The strategy for making Kdm7a cKO mice is shown (A). The genotypes of the mutant mice were confirmed by genotyping PCR (B). Lables 1: Kdm7a$^{fx/fx}$; 2: Kdm7a cKO; 3: Positive control; 4: Negative control. (C-J) Measurements of body length (total length minus that of the tail), weight and bone length. Data are presented as box-and-whiskers plots, n=10. Student's t test was performed for statistical analyses, ns: no significance.

## Figure S3

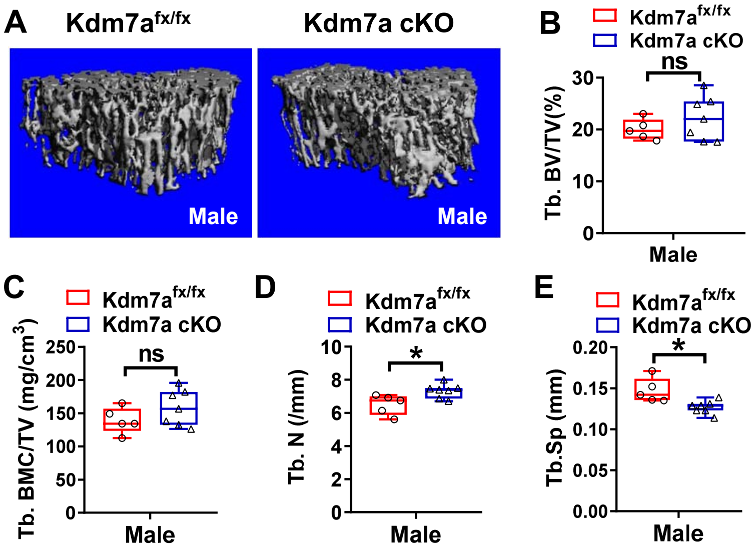


**Figure S3. Cancellous bone mass was not changed in 12-week-old Kdm7a mice.** μCT analyses were performed on the cancellous bone architecture in the proximal tibial metaphysis of 12-week-old male mice. The reconstruction images are shown (A). Histomorphometric parameters were analyzed on cancellous bone (B-E). Data are presented as box-and-whiskers plots, n=5~7. Comparisons were conducted using Student's t test. *p < 0.05, ns: no significance.

## Figure S4

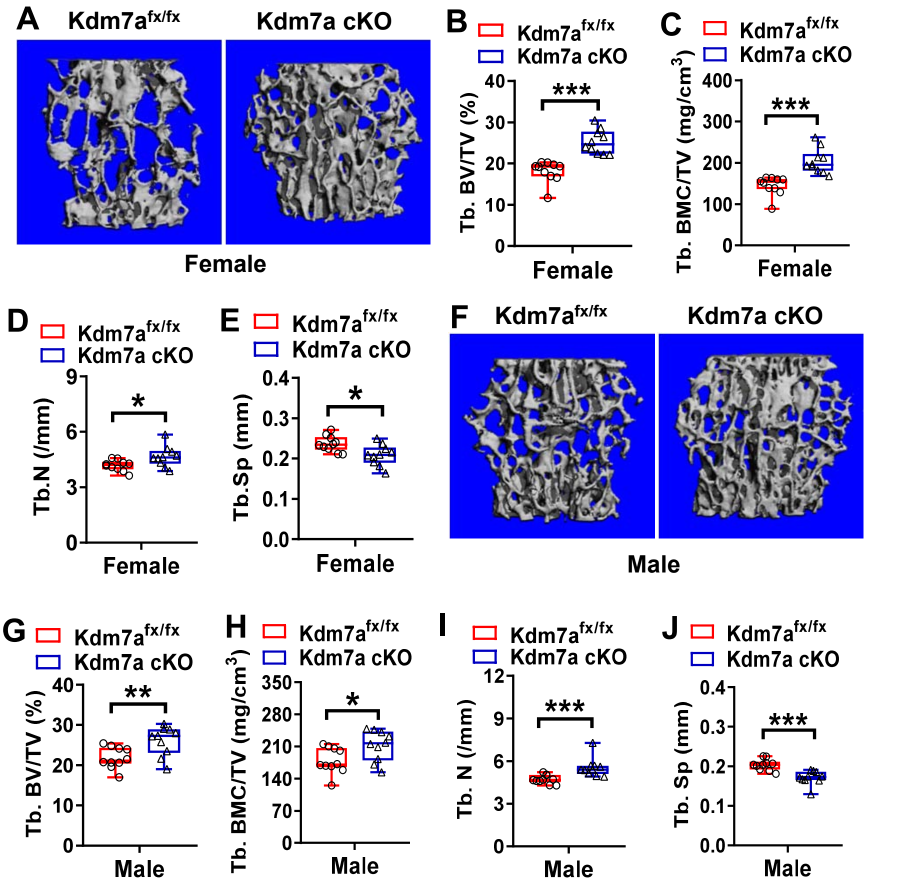


**Figure S4. Deletion of Kdm7a in osteoprogenitor cells promoted bone mass accrual in lumbar vertebrae of mice.** μCT analyses were performed on the bone architecture in the L2 Lumbar vertebrae of 24-week-old female (A-E) and male (F-J) mice. The reconstruction images are shown (A: female; F: male). Histomorphometric parameters were analyzed on cancellous bone in female (B-E) and male (G-J) mice. Data are presented as box-and-whiskers plots, n=10. Student's t test was performed for statistical analyses. *$p < 0.05$, **$p < 0.01$, ***$p < 0.001$.

## Figure S5

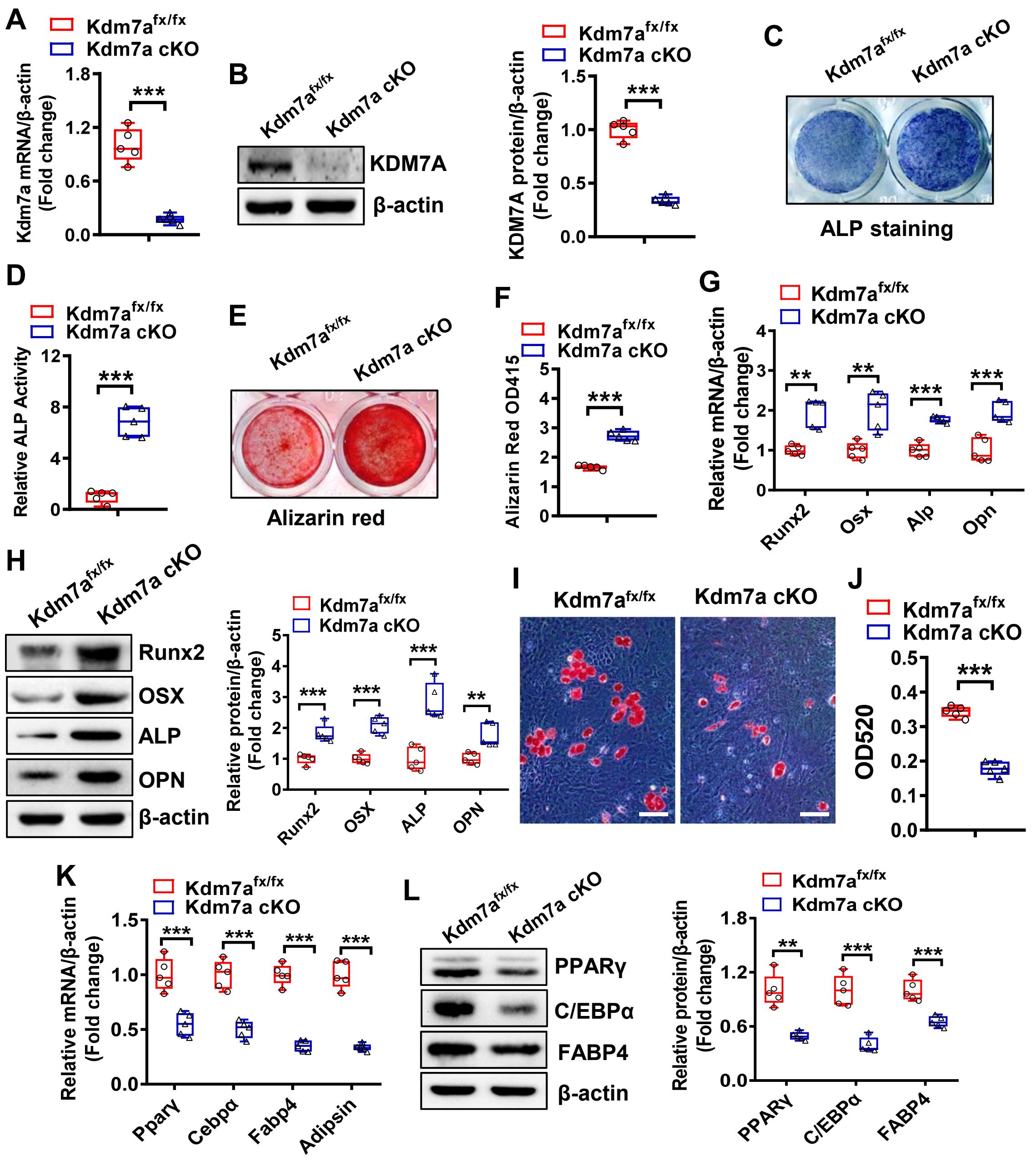


**Figure S5. Differentiation was dysregulated in pre-osteoblastic cells from Kdm7a cKO mice.** RT-qPCR and Western blotting were performed to verify the deletion of Kdm7a in calvarial pre-osteoblastic cells from the Kdm7a cKO mice (A, B). Fourteen days after osteogenic treatment, ALP staining was performed (C) and ALP activity (D) was measured. Twenty one days after osteogenic induction, alizarin red staining was performed (E) and the intensity of staining was quantified (F). The mRNA (G) and protein (H) levels of osteogenic factors were detected 72 h after induction by RT-qPCR and Western blotting, respectively. Five days after adipogenic induction, oil red O staining was performed (I) and the quantity of dye accumulation in the cells was measured (J). The mRNA (K) and protein (L) levels of adipogenic factors were examined 48 h and 72 h respectively after induction. Image scale in (I): 100 μm. Data are presented as box-and-whiskers plots, n=5. Comparisons were conducted using Student's t test. $^{**}p < 0.01$, $^{***}p < 0.001$.

Figure S6

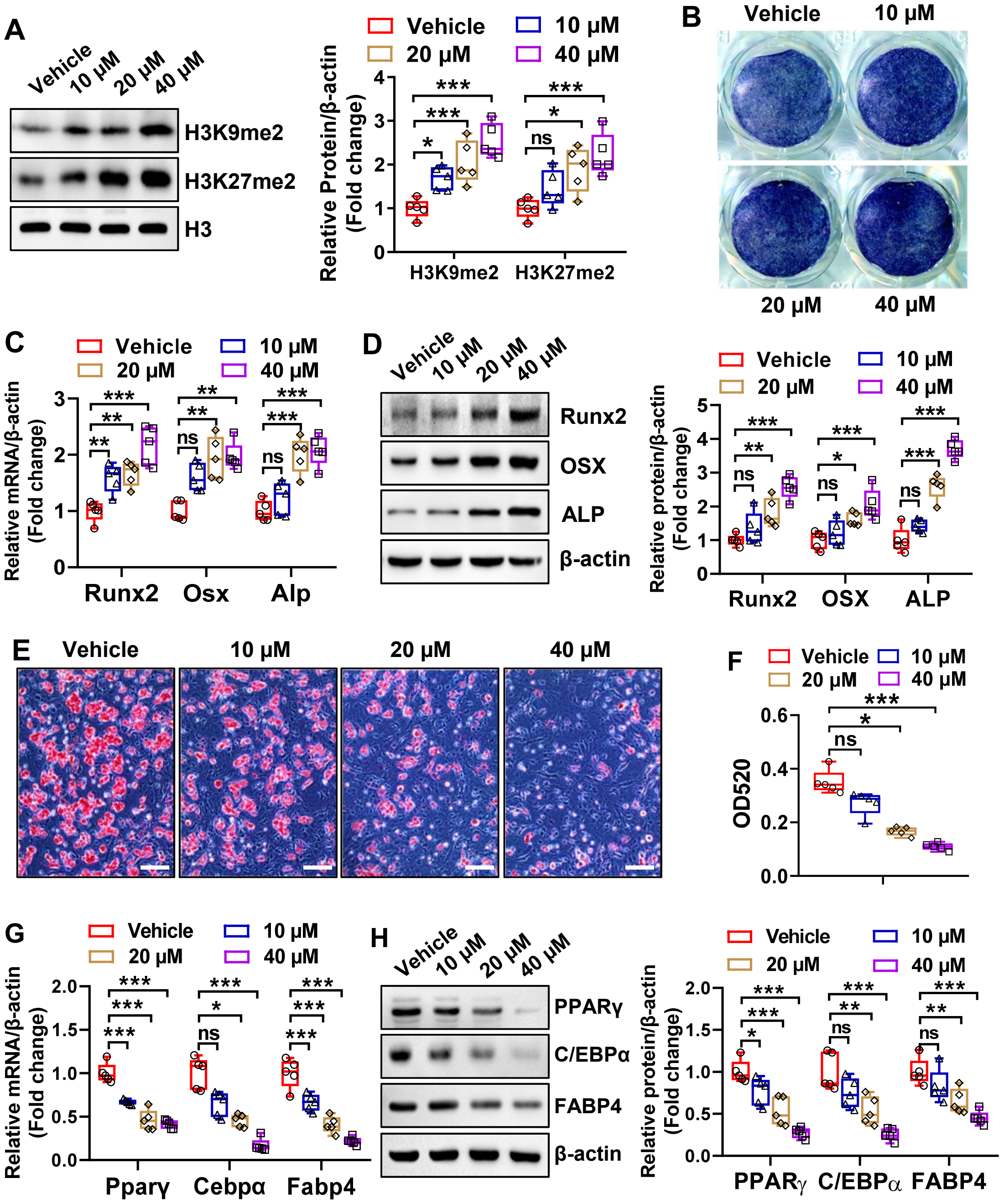


**Figure S6. TC-E 5002 treatment promoted osteoblast differentiation and blocked adipocyte formation from BMSCs.** BMSCs was treated with different doses of TC-E 5002 and the levels of H3K9me2 and H3K27me3 were examined using Western blotting (A). Fourteen days after osteogenic treatment, ALP staining was performed (B). The mRNA (C) and protein (D) levels of osteogenic factors were measured 72 h after induction by RT-qPCR and Western blotting, respectively. Five days after adipogenic induction, oil red O staining was performed (E) and the quantity of dye accumulation in the cells was measured (F). The mRNA (G) and protein (H) levels of adipogenic factors were examined 48 h and 72 h respectively after induction. Image scale in (E): 100 μm. Data are presented as box-and-whiskers plots, n=5. Comparisons were conducted using One-way ANOVA followed by Dunnett'stest.*p < 0.05, **p < 0.01, ***p < 0.001, ns: no significance.

**Figure S7**

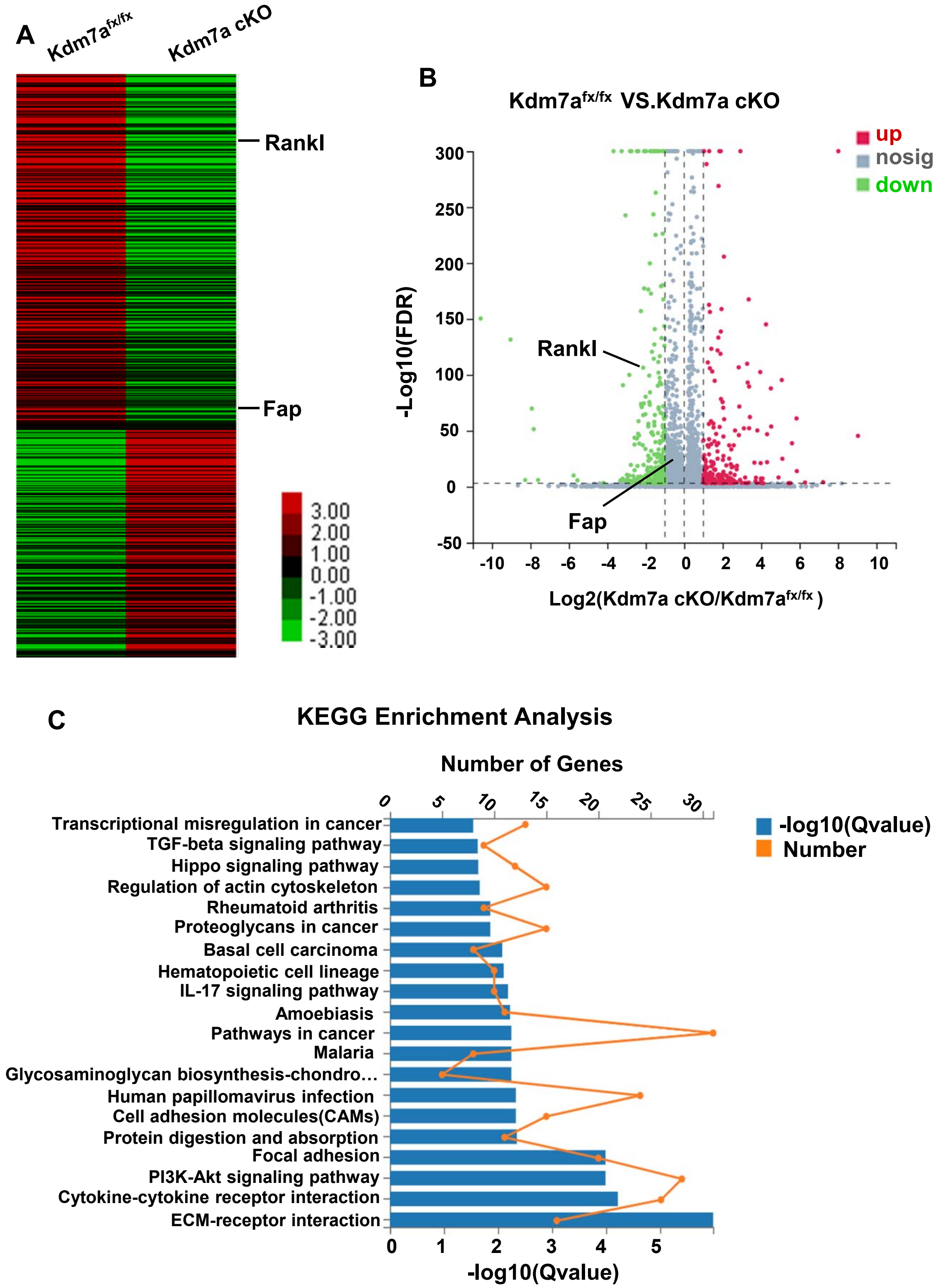


**Figure S7. RNA-seq revealed differentially expressed genes in calvarial pre-osteoblastic cells derived from Kdm7a cKO and Kdm7a$^{fx/fx}$ mice.** The differentially expressed genes in calvarial pre-osteoblastic cells derived from Kdm7a cKO and Kdm7a$^{fx/fx}$ control mice are shown (A). Q adjust and fold change >2 are set as restrictive conditions to identify the differentially expressed genes (B). KEGG enrichment analysis of related pathways were done and the top-ranking enriched pathways are shown (C).

## Figure S8

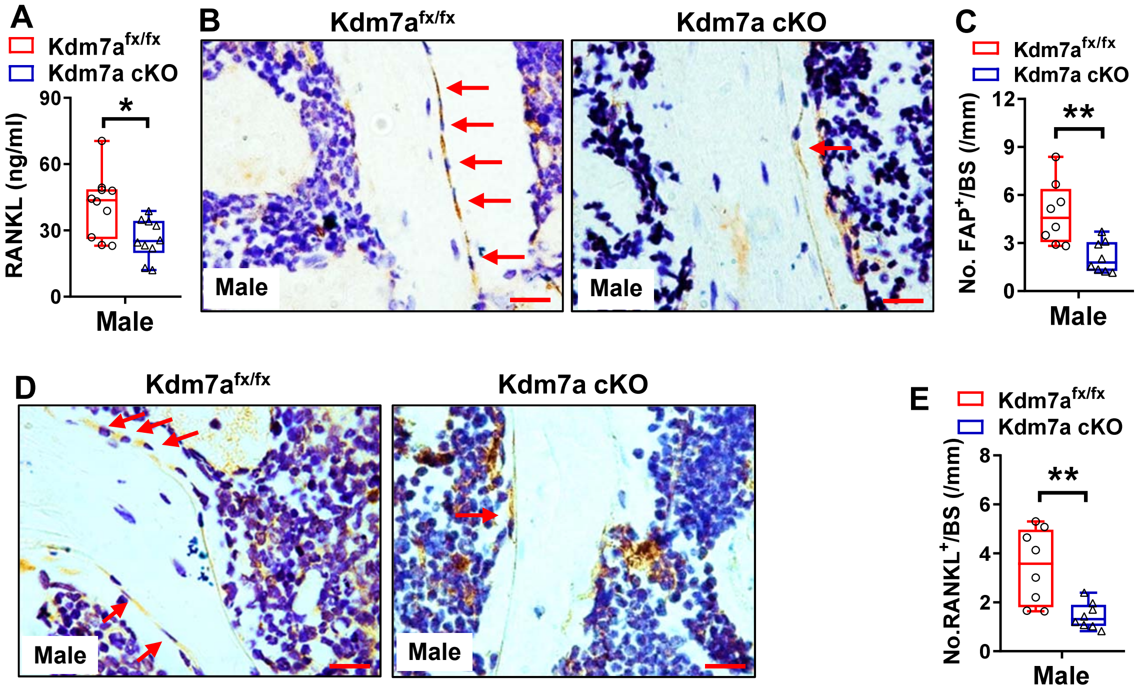


**Figure S8. Kdm7a deletion in osteoprogenitor cells downregulated FAP and RANKL expression in male mice.** Serum RANKL levels were measured in 24-week-old male mice using ELISA (A). IHC staining of FAP and RANKL was performed (B, D). FAP-positive cells and RANKL-positive cells on trabeculae were counted (C, E) . Image scale in (B,D): 20 μm. Data are presented as box-and-whiskers plots, n=10 in (A); n=8 in (C, E). Comparisons were conducted using Student's t test. *$p < 0.05$, **$p < 0.01$.

## Figure S9

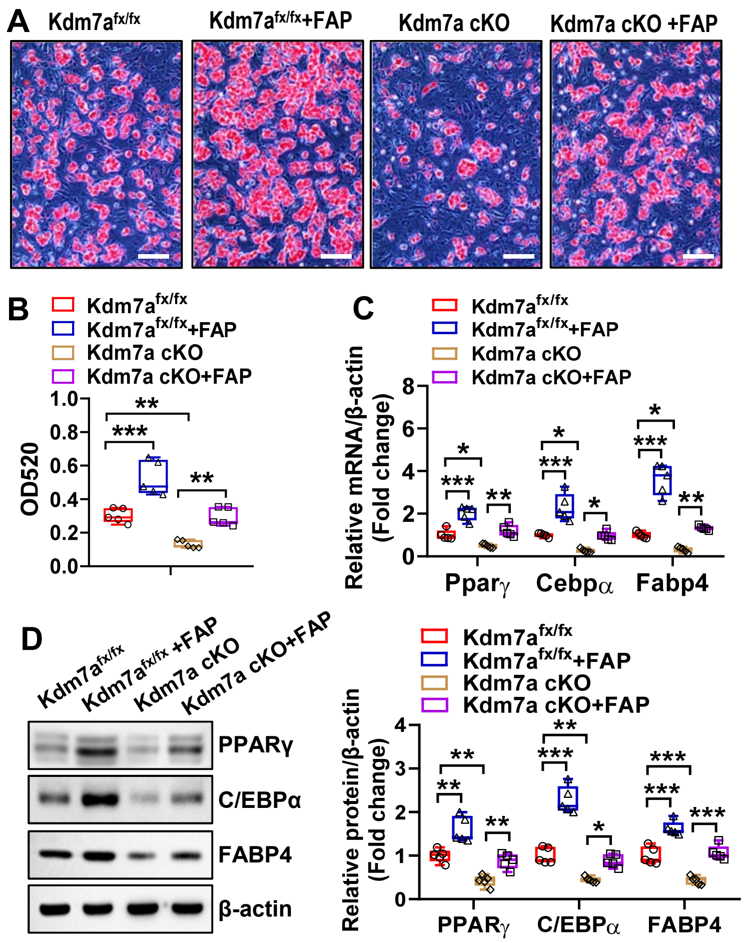


**Figure S9. FAP attenuated Kdm7a deletion-induced dysregulation of adipogenic differentiation.** Primary BMSCs were cultured, and treated with recombinant FAP followed by adipogenic induction. Oil red O staining was performed 5 days after adipogenic induction and the quantity of oil red O accumulation in cells was measured (A, B). The mRNA (C) and protein (D) levels of adipogenic factors were measured 72 h after adipogenic induction. Image scale in (A): 100 μm. Data are presented as box-and-whiskers plots, n=5. Comparisons were conducted using two-way ANOVA followed by Tukey's test. $^{*}p < 0.05$, $^{**}p < 0.01$, $^{***}p < 0.001$.

**Supplemental Table S1. Primers used for Genotyping, RT-PCR and ChIP assays**

| Genes | Forward primer sequences | Reverse primer sequences |
|---|---|---|
| Kdm7a genotyping | CAATGCCAGATAAGTGCTGGCTAG | CTGGCATTACTGACCTAAGCGC |
| Osx-Cre genotyping | TACCAGAAGCGACCACTTGAGC | GCACACAGACAGGAGCATCTTC |
| PPARγ | CTTGACAGGAAAGACAACGG | GCTTCTACGGATCGAAACTG |
| C/EBPα | CTGATTCTTGCCAAACTGAG | GAGGAAGCTAAGACCCACTAC |
| Fabp4 | AAATCACCGCAGACGACAGG | GGCTCATGCCCTTTCATAAAC |
| adipsin | TGATGTGTGCAGAGAGCAAC | CGTAACCACACCTTCGACTG |
| Runx2 | TCCTGTAGATCCGAGCACCA | CTGCTGCTGTTGTTGCTGTT |
| Alp | CCAGAAAGACACCTTGACTGTGG | TCTTGTCCGTGTCGCTCACCAT |
| Osterix | GGCTTTTCTGCGGCAAGAGGTT | CGCTGATGTTTGCTCAAGTGGTC |
| Osteocalcin | GCAATAAGGTAGTGAACAGACTCC | CCATAGATGCGTTTGTAGGCGG |
| Kdm7a | GATCGAGTGCGATGTCTGCAAG | TTAAGGAAGAGCCGTGTAGAGCTG |
| Rankl | GTGAAGACACACTACCTGACTCC | GCCACATCCAACCATGAGCCTT |
| Fap | CCGCGTAACACAGGATTCACTG | CACACTTCTTGCTCGGAGGAGA |
| β-actin | AAGACCTCTATGCCAACACAG | GGAGGAGCAATGATCTTGATC |
| Rankl (ChIP) | AGGCTATATTTGGAGGGATAACTG | CTCTAGTGAACTCCGTCCGAAGCC |
| Rankl (ChIP) Ctrl | GGCTTAGATGATCCTTCTATTGGC | CACAGAAAGATGTATGCCGTTATT |
| Fap (ChIP) | CCCAAACATTTGATATGTACAATG | ATCACTCGAGTCACTTTAGTAACA |
| Fap (ChIP) Ctrl | GGCAGAAAAGCTAATGTGCTGCAC | TAACTTTTGGGAGGGTGAGTTTTT |

**Supplemental Table S2. Primary antibodies for Western blotting**

| Antibody | Catalog number | Brand |
|---|---|---|
| anti-Runx2 | ab192256 | Abcam (Cambridge, MA, USA) |
| anti-low-density lipoprotein receptor-related protein 6 (LRP6) | ab134146 | |
| anti-KDM7A | A8266 | Abclonal (Wuhan, China) |
| anti-β-actin | AC026 | |
| anti-nuclear factor of activated T cells 1 (NFATc1) | A1539 | |
| anti-cathepsin K (CTSK) | A1782 | |
| anti-osterix | DF7713 | Affinity (Changzhou, China) |
| anti-non-phospho-β-catenin | #8814 | Cell Signaling Technology (Danvers, MA, USA) |
| anti-C/EBPα | #8178 | |
| anti-PPARγ | #2443 | |
| anti-phospho-LRP6 (Ser1490) | #2568 | |
| anti-ALP | (ET1601-21) | Huabio (Hangzhou, China) |
| anti-fatty acid binding protein 4 (FABP4) | 67167-1-Ig | Proteintech (Wuhan, China) |
| anti-osteopontin | 22952-1-AP | |
| anti-glycogen synthase kinase 3β (GSK3β) | 24198-1-AP | |
| anti-phospho-GSK3β (S9) | 67558-1-Ig | |
| anti-β-catenin | 66379-1-Ig | |
| anti-RANKL | WL00285 | Wanleibio (Shenyang, China) |
| anti-fibroblast activation protein α (FAP) | WL04890 | |
