# Supplementary material for: Histone demethylase KDM7A regulates bone homeostasis through balancing osteoblast and osteoclast differentiation
Source: Cell Death Dis. 2024 Feb 12;15(2):136. doi: 10.1038/s41419-024-06521-z (PMC10861515; doi:10.1038/s41419-024-06521-z)
Supplement: Supplementary file 3 — Original WB images [file 41419_2024_6521_MOESM3_ESM.pdf]

Figure 1B

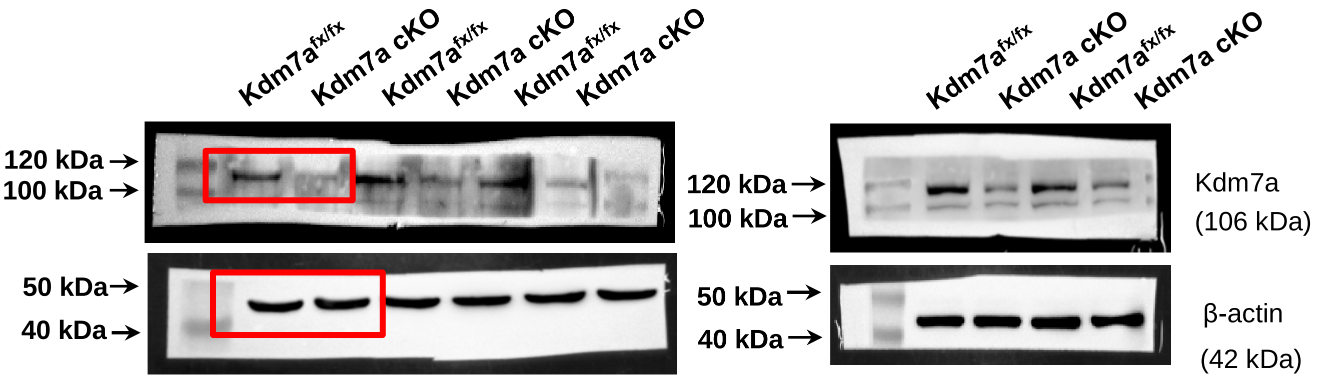

Figure 1C

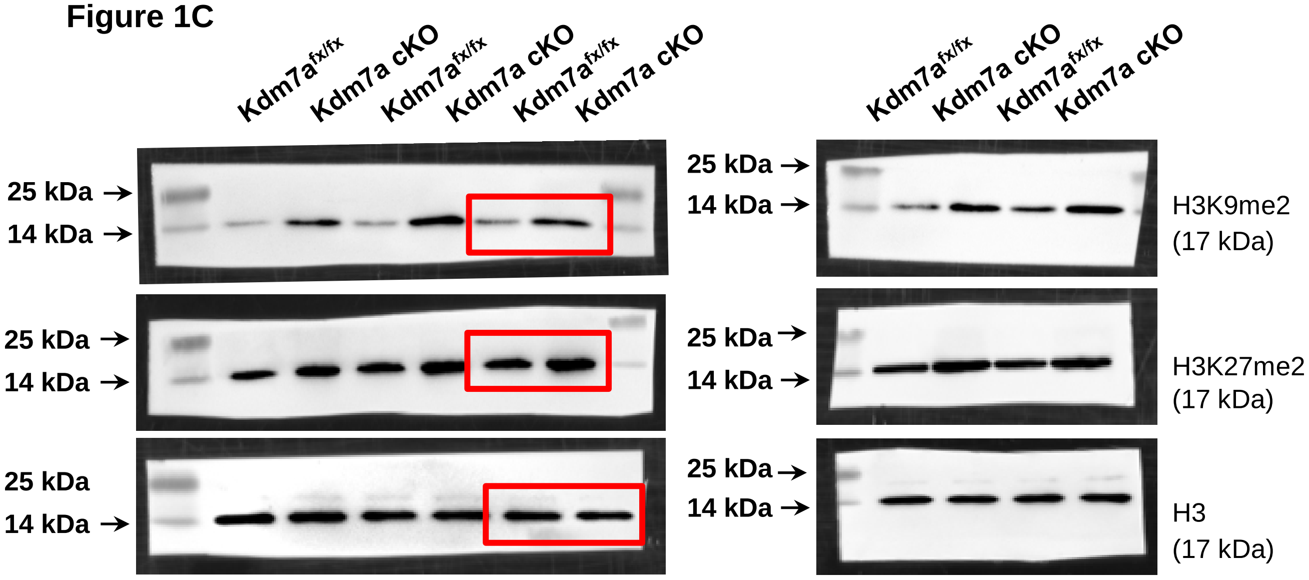

Figure 4C

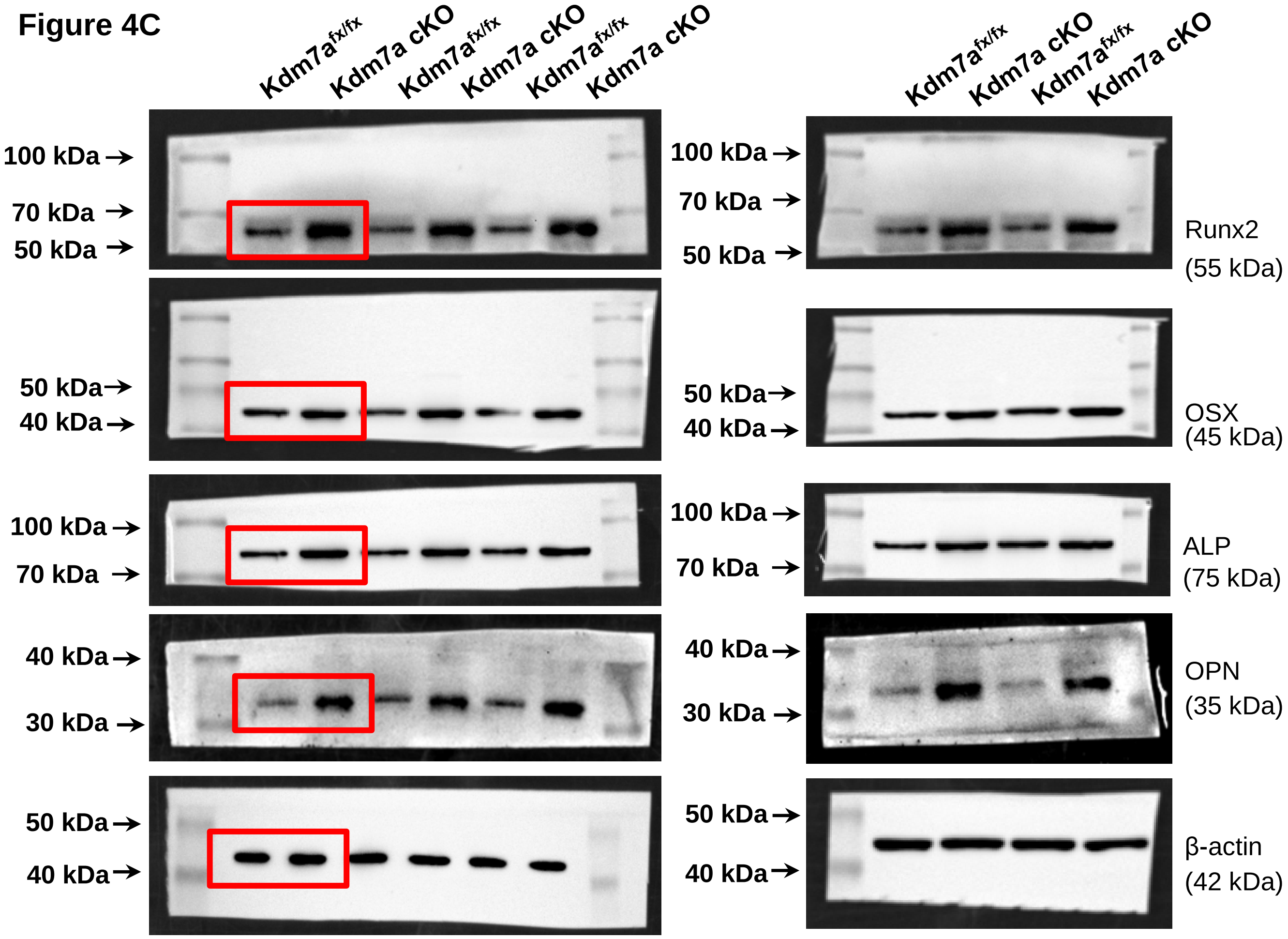

Figure 4G

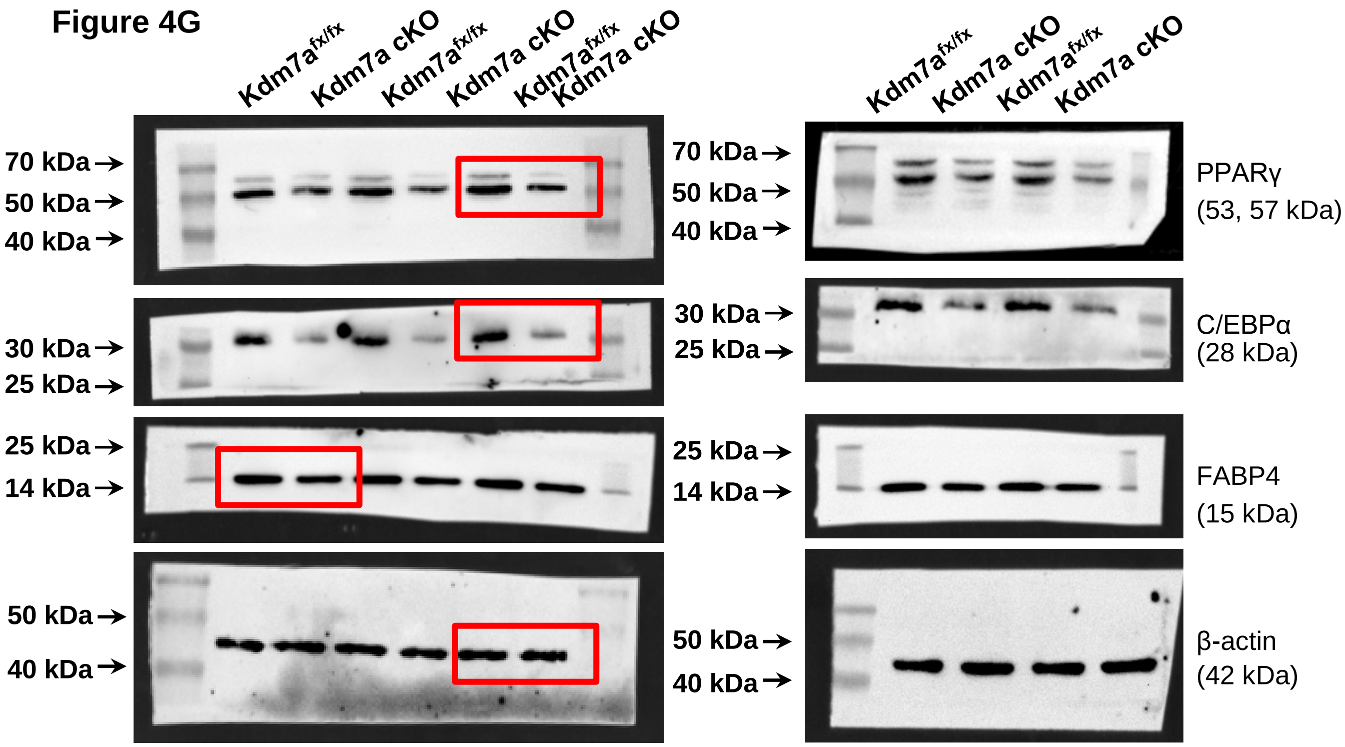

Figure 5D

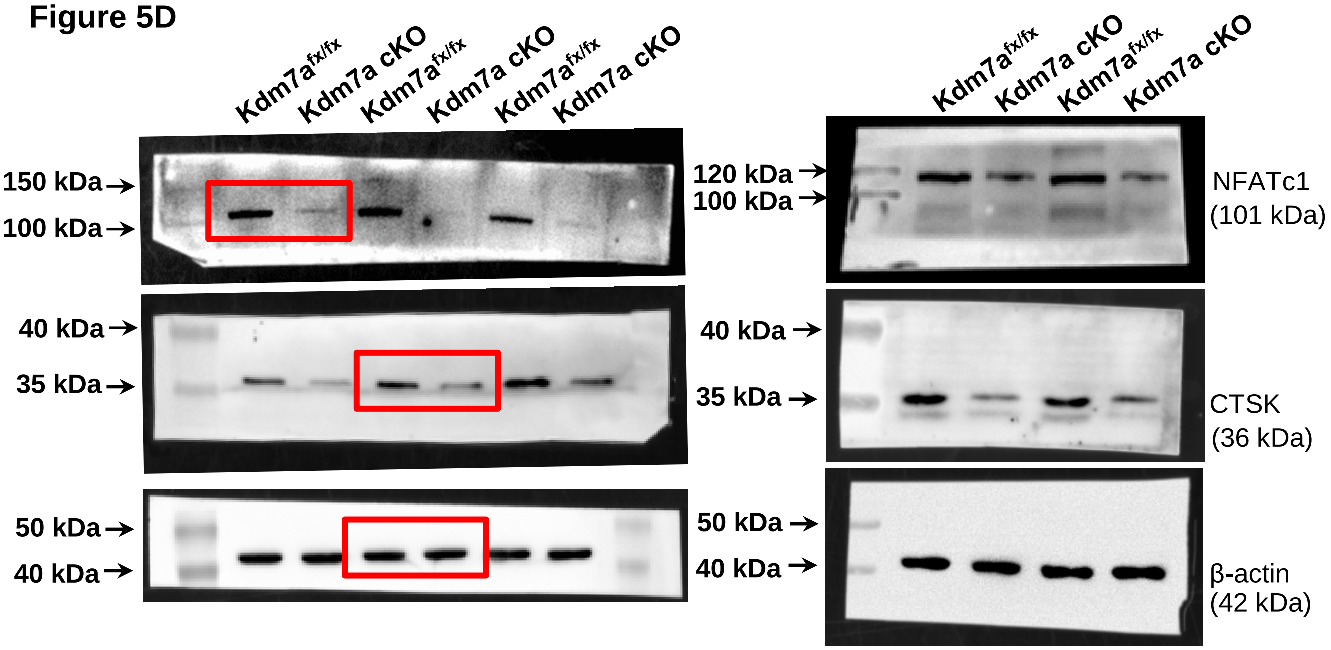

Figure 5H

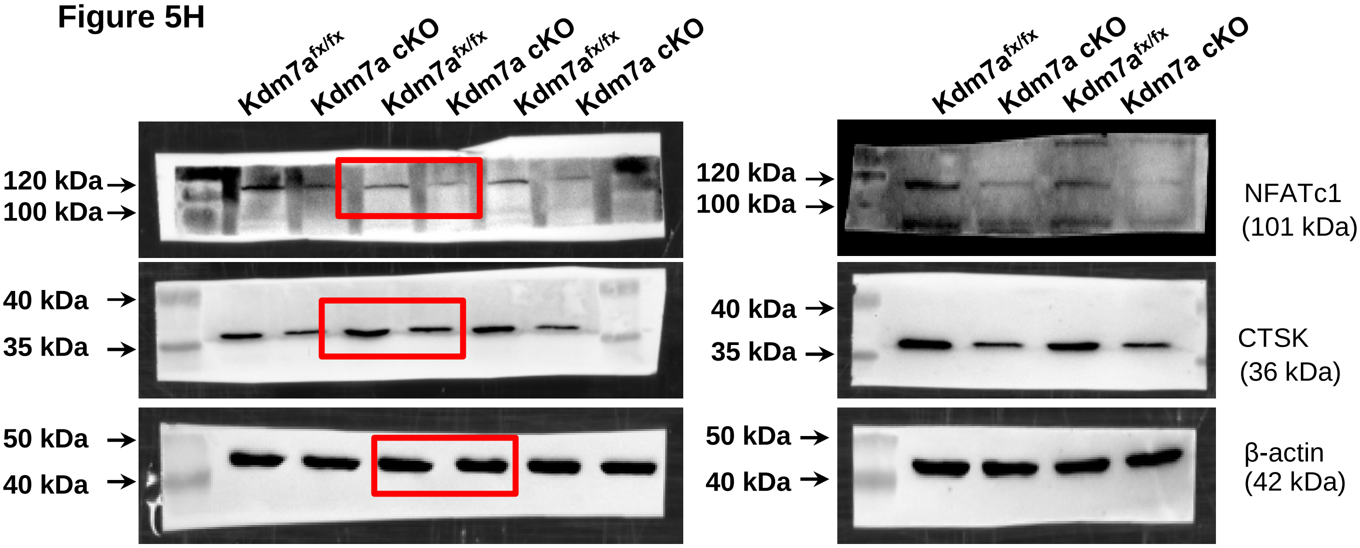

Figure 5L

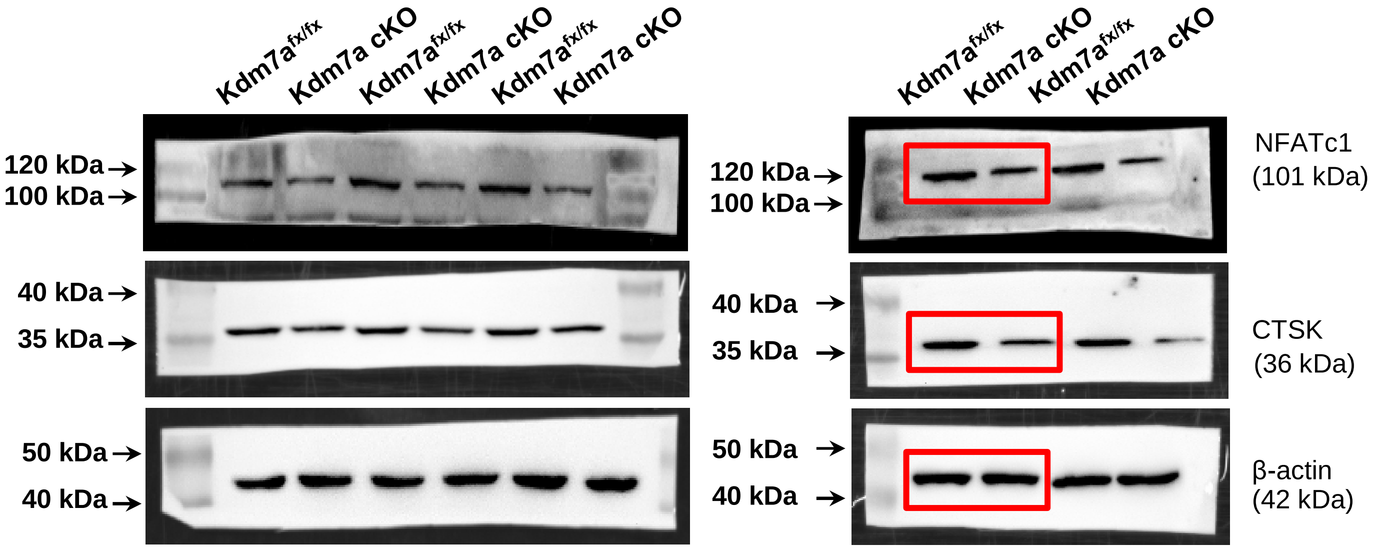

Kdm7afx/fx
Kdm7a cKO
Kdm7afx/fx
Kdm7a cKO
Kdm7afx/fx
Kdm7a cKO
120 kDa
100 kDa
40 kDa
35 kDa
50 kDa
40 kDa
Kdm7afx/fx
Kdm7a cKO
Kdm7afx/fx
Kdm7a cKO
120 kDa
100 kDa
40 kDa
35 kDa
50 kDa
40 kDa
NFATc1
(101 kDa)
CTSK
(36 kDa)
β-actin
(42 kDa)

Figure 6B

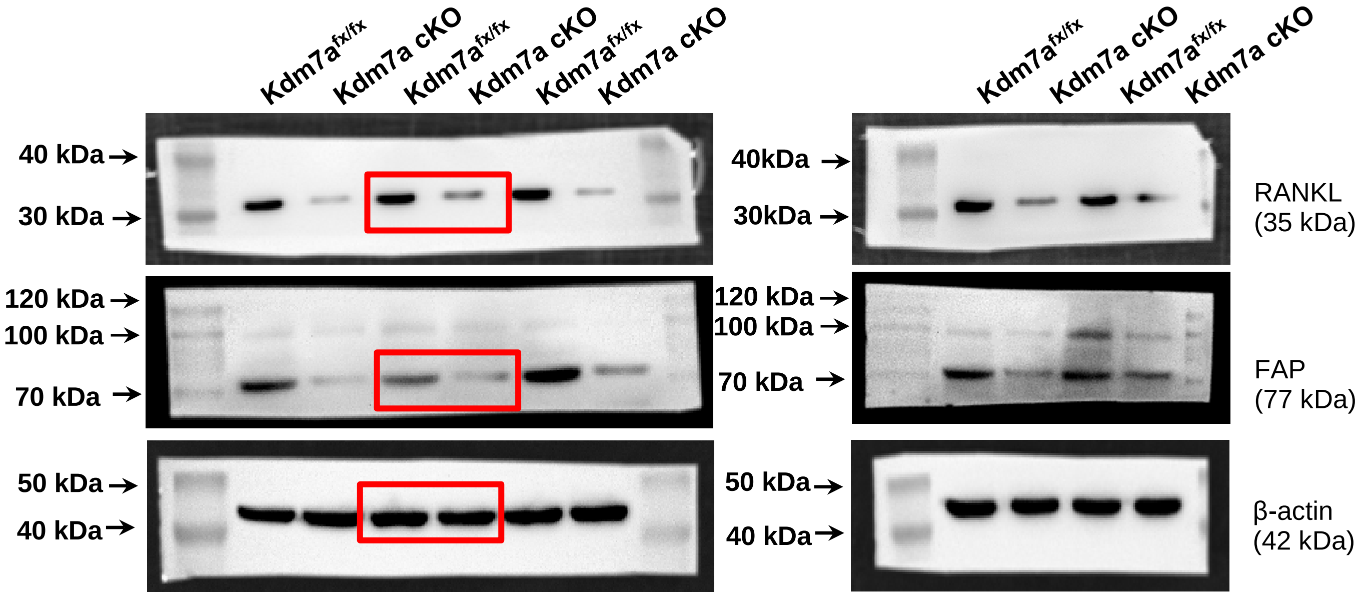

Kdm7a fx/fx
Kdm7a cKO
Kdm7a fx/fx
Kdm7a cKO
Kdm7a fx/fx
Kdm7a cKO
Kdm7a fx/fx
Kdm7a cKO
Kdm7a fx/fx
Kdm7a cKO
40 kDa
30 kDa
40kDa
30kDa
RANKL
(35 kDa)
120 kDa
100 kDa
70 kDa
120 kDa
100 kDa
70 kDa
FAP
(77 kDa)
50 kDa
40 kDa
50 kDa
40 kDa
β-actin
(42 kDa)

Figure 7G

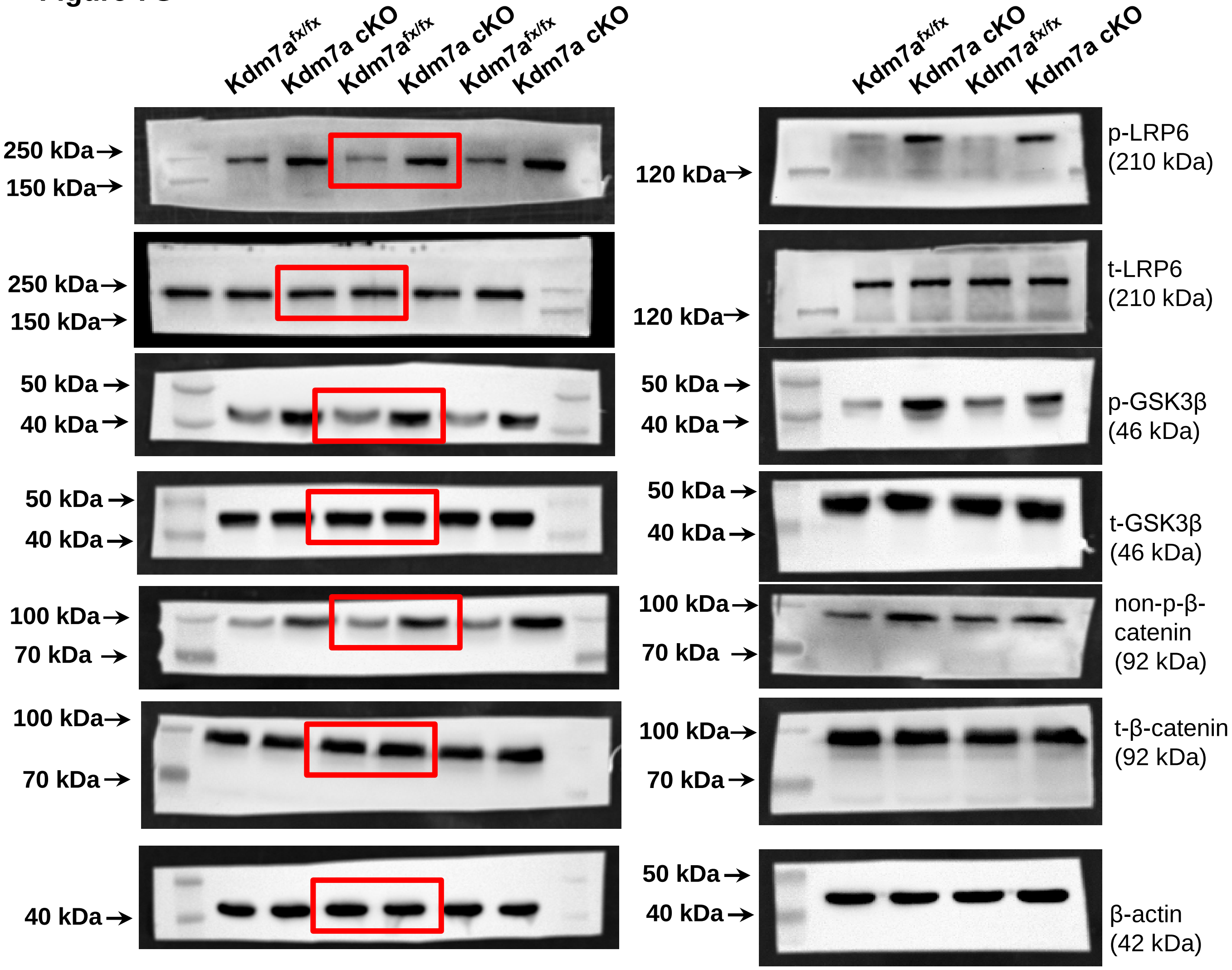

Kdm7a$^{fx/fx}$
Kdm7a cKO
Kdm7a$^{fx/fx}$
Kdm7a cKO
Kdm7a$^{fx/fx}$
Kdm7a cKO
Kdm7a$^{fx/fx}$
Kdm7a cKO
Kdm7a$^{fx/fx}$
Kdm7a cKO
250 kDa→
150 kDa→
120 kDa→
p-LRP6 (210 kDa)
250 kDa→
150 kDa→
120 kDa→
t-LRP6 (210 kDa)
50 kDa→
40 kDa→
50 kDa→
40 kDa→
p-GSK3β (46 kDa)
50 kDa→
40 kDa→
50 kDa→
40 kDa→
t-GSK3β (46 kDa)
100 kDa→
70 kDa→
100 kDa→
70 kDa→
non-p-β-catenin (92 kDa)
100 kDa→
70 kDa→
100 kDa→
70 kDa→
t-β-catenin (92 kDa)
40 kDa→
50 kDa→
40 kDa→
β-actin (42 kDa)

Figure 8C

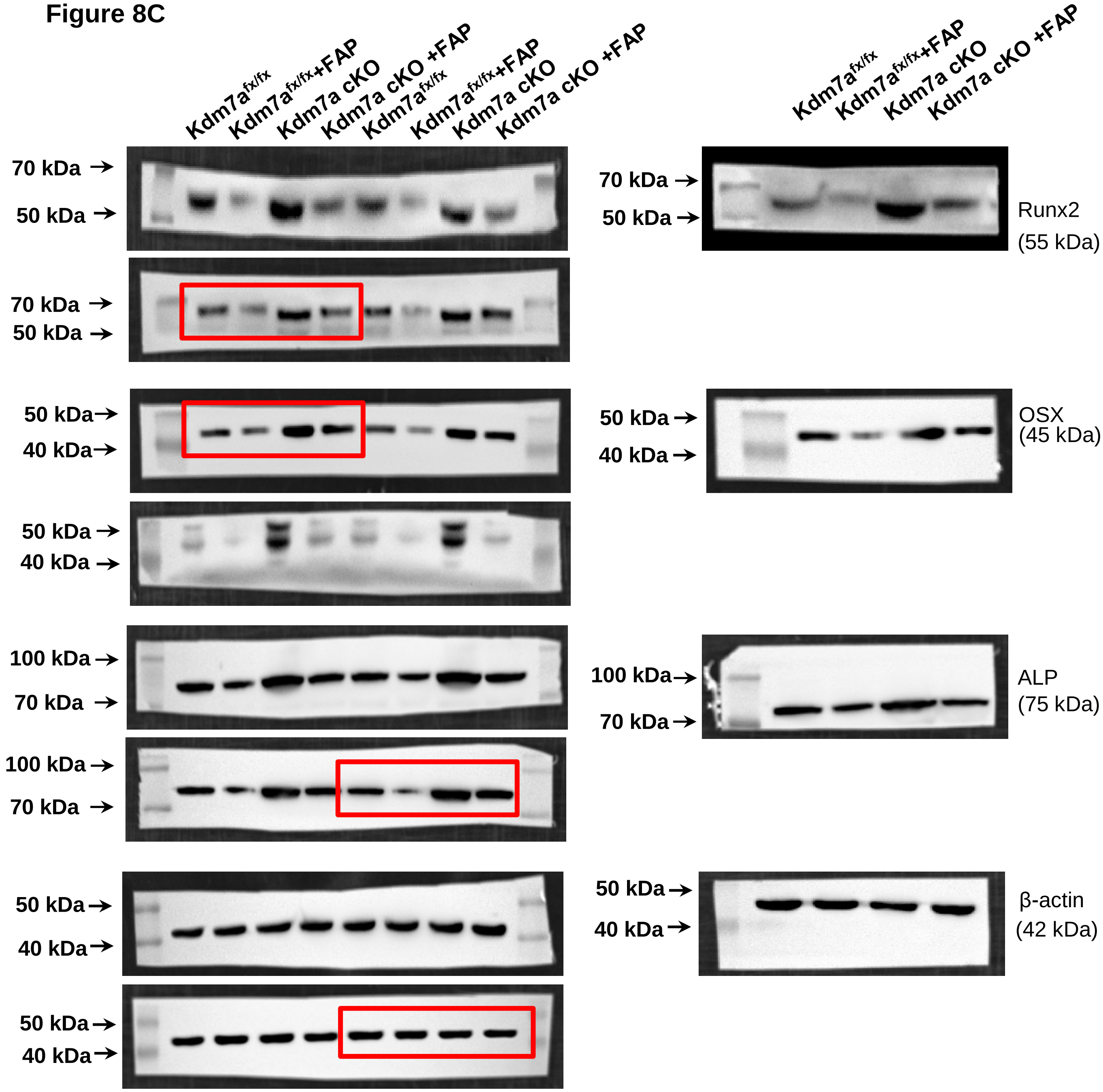

Kdm7a$^{fx/fx}$
Kdm7a$^{fx/fx}$+FAP
Kdm7a cKO
Kdm7a cKO +FAP
Kdm7a$^{fx/fx}$
Kdm7a$^{fx/fx}$+FAP
Kdm7a cKO
Kdm7a cKO +FAP
Kdm7a$^{fx/fx}$
Kdm7a$^{fx/fx}$+FAP
Kdm7a cKO
Kdm7a cKO +FAP
70 kDa
50 kDa
40 kDa
100 kDa
Runx2
(55 kDa)
OSX
(45 kDa)
ALP
(75 kDa)
β-actin
(42 kDa)

Figure 8D

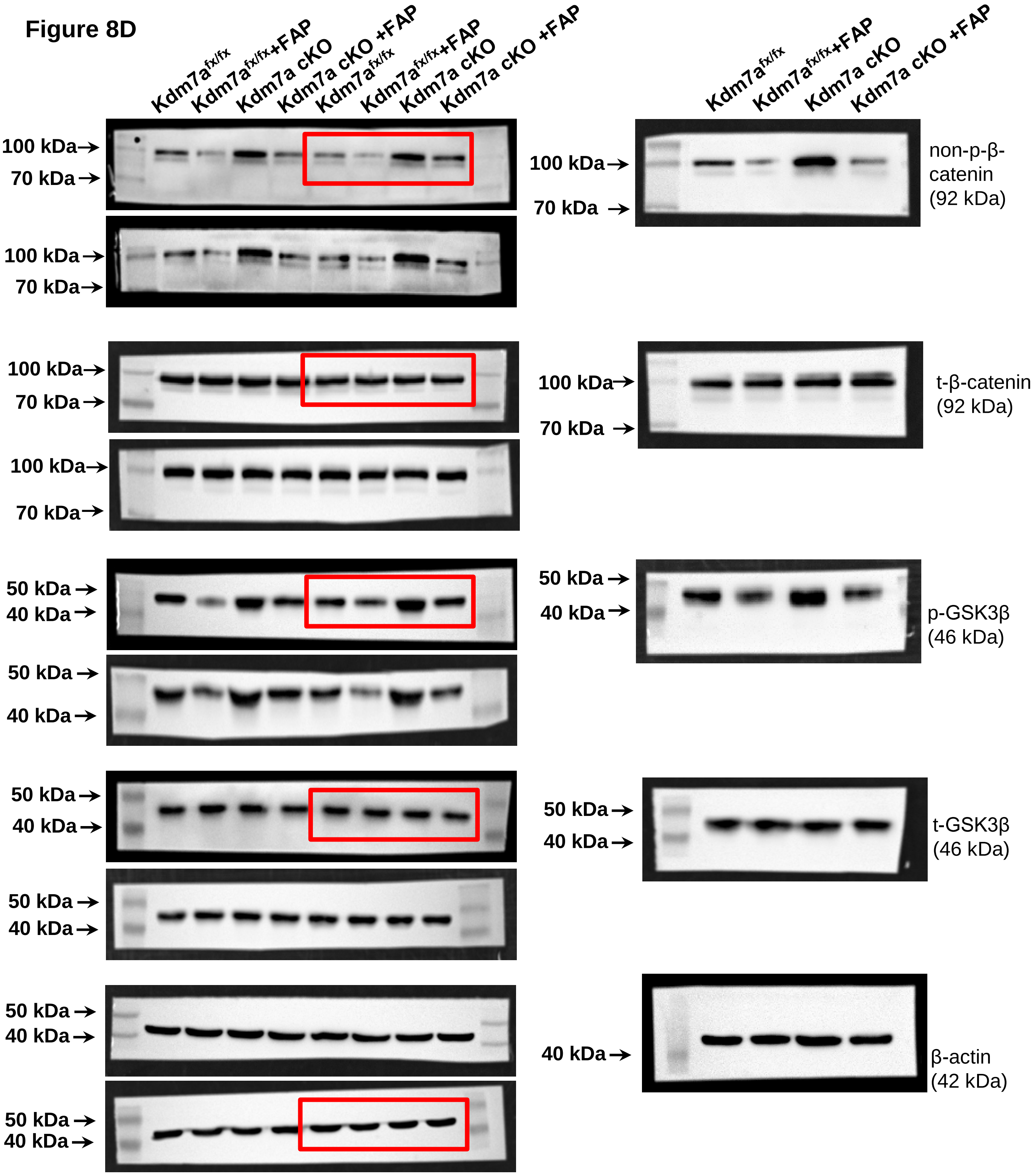
Kdm7a fx/fx
Kdm7a fx/fx +FAP
Kdm7a cKO
Kdm7a cKO +FAP
Kdm7a fx/fx
Kdm7a fx/fx +FAP
Kdm7a cKO
Kdm7a cKO +FAP
Kdm7a fx/fx
Kdm7a fx/fx +FAP
Kdm7a cKO
Kdm7a cKO +FAP
100 kDa
70 kDa
50 kDa
40 kDa
non-p-β-catenin (92 kDa)
t-β-catenin (92 kDa)
p-GSK3β (46 kDa)
t-GSK3β (46 kDa)
β-actin (42 kDa)

Figure S5B

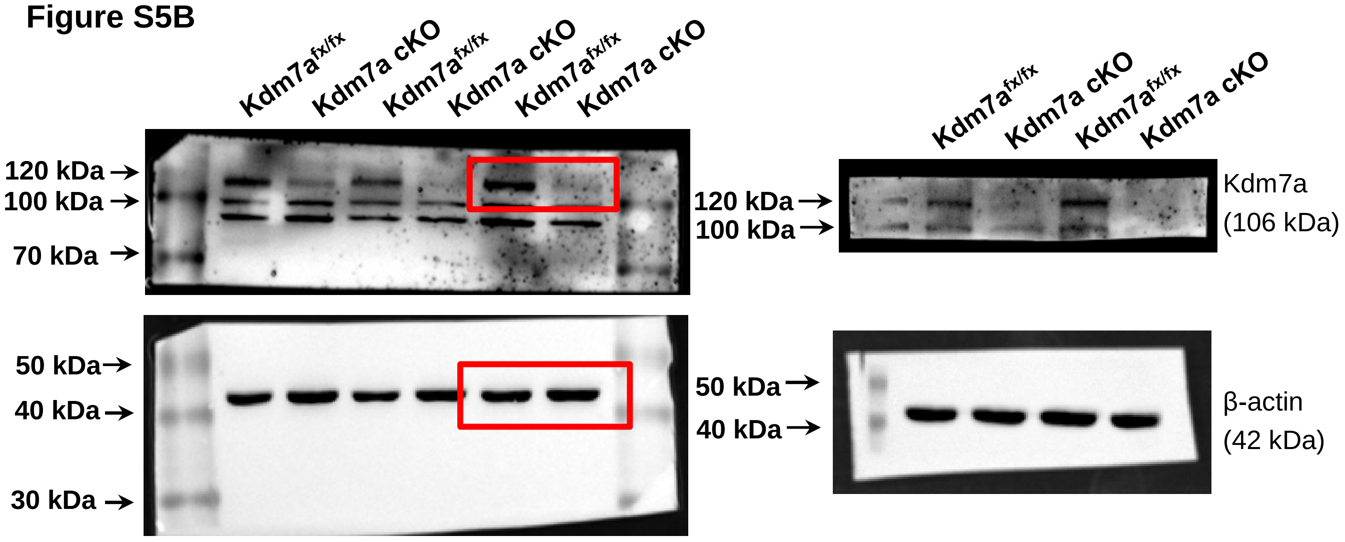

Figure S5H

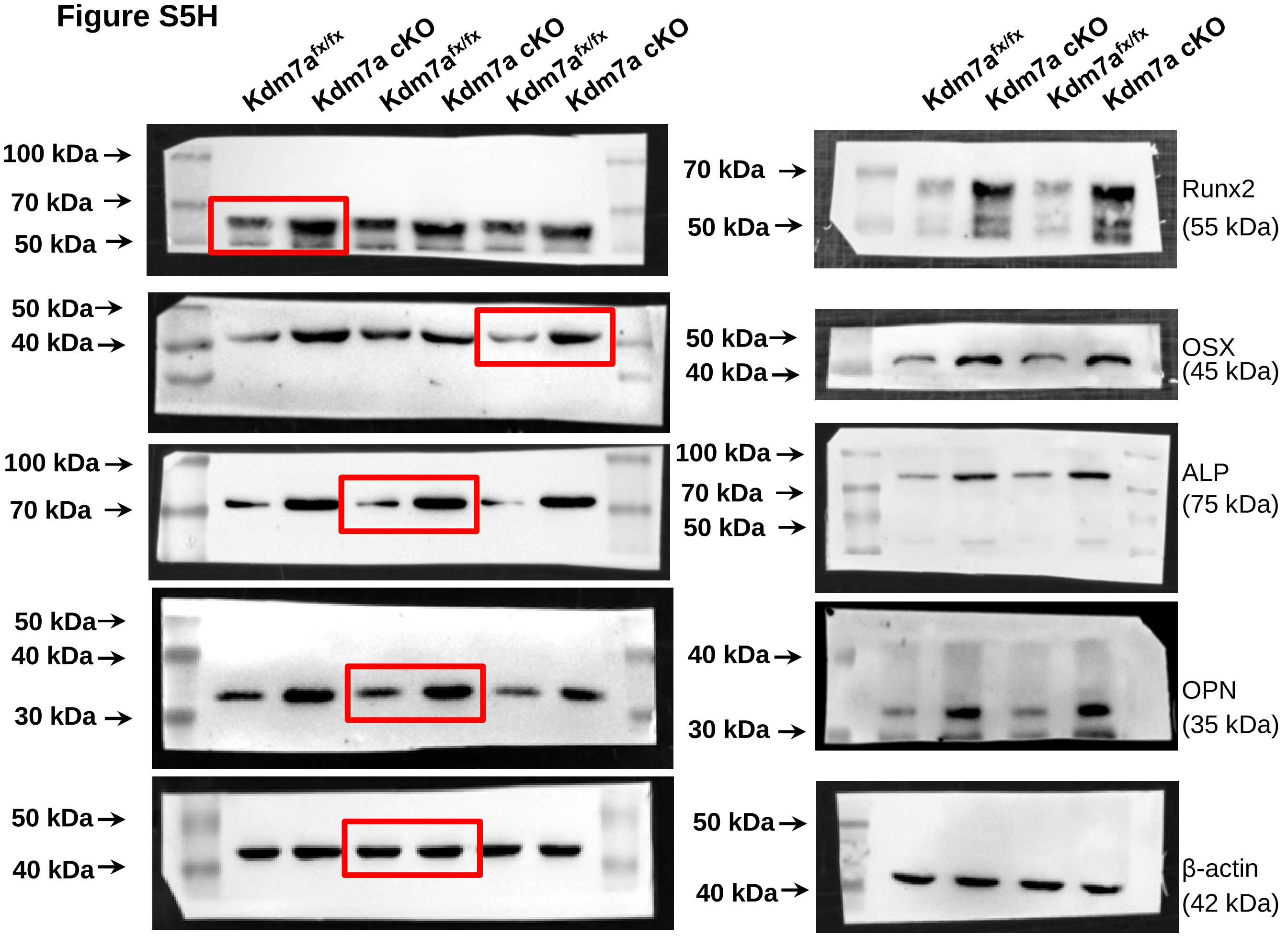

Figure S5L

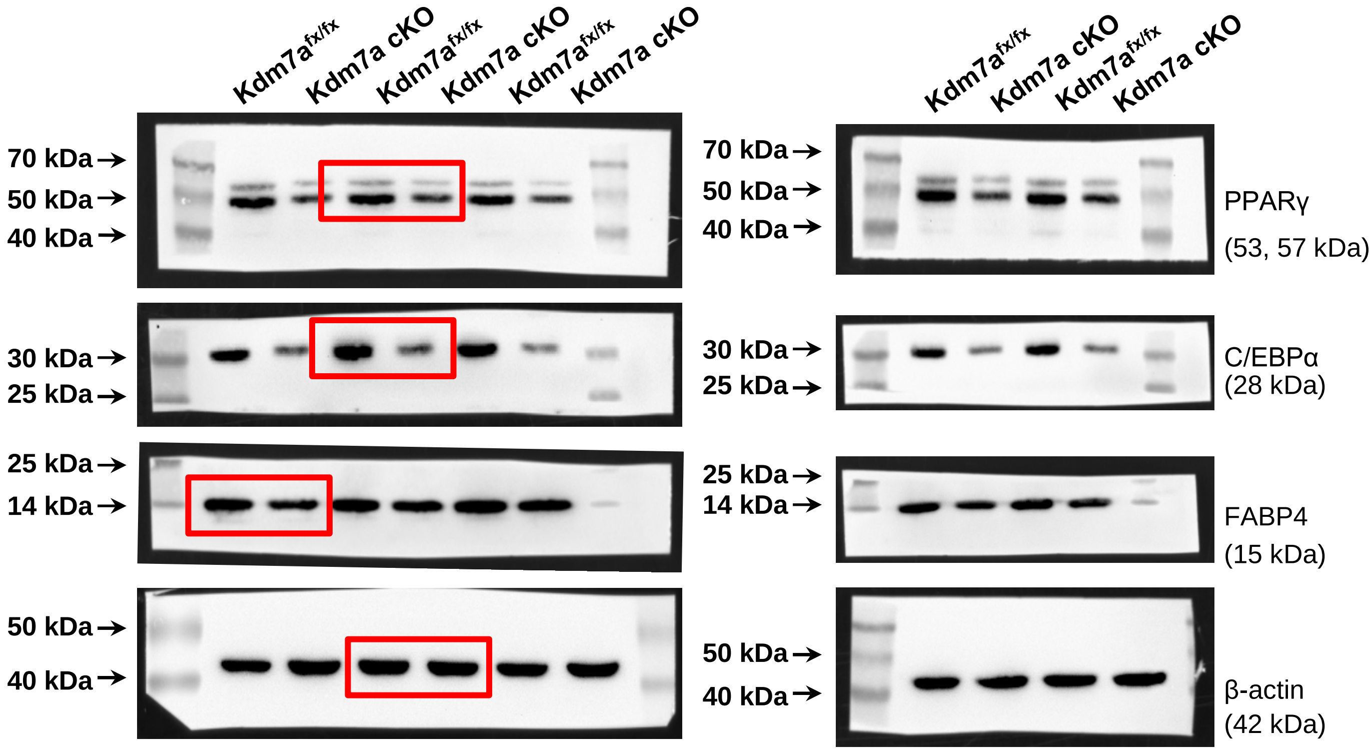

Kdm7afx/fx
Kdm7a cKO
Kdm7afx/fx
Kdm7a cKO
Kdm7afx/fx
Kdm7a cKO
Kdm7afx/fx
Kdm7a cKO
Kdm7afx/fx
Kdm7a cKO
70 kDa
50 kDa
40 kDa
PPARγ
(53, 57 kDa)
30 kDa
25 kDa
C/EBPα
(28 kDa)
25 kDa
14 kDa
FABP4
(15 kDa)
50 kDa
40 kDa
β-actin
(42 kDa)

Figure S6A

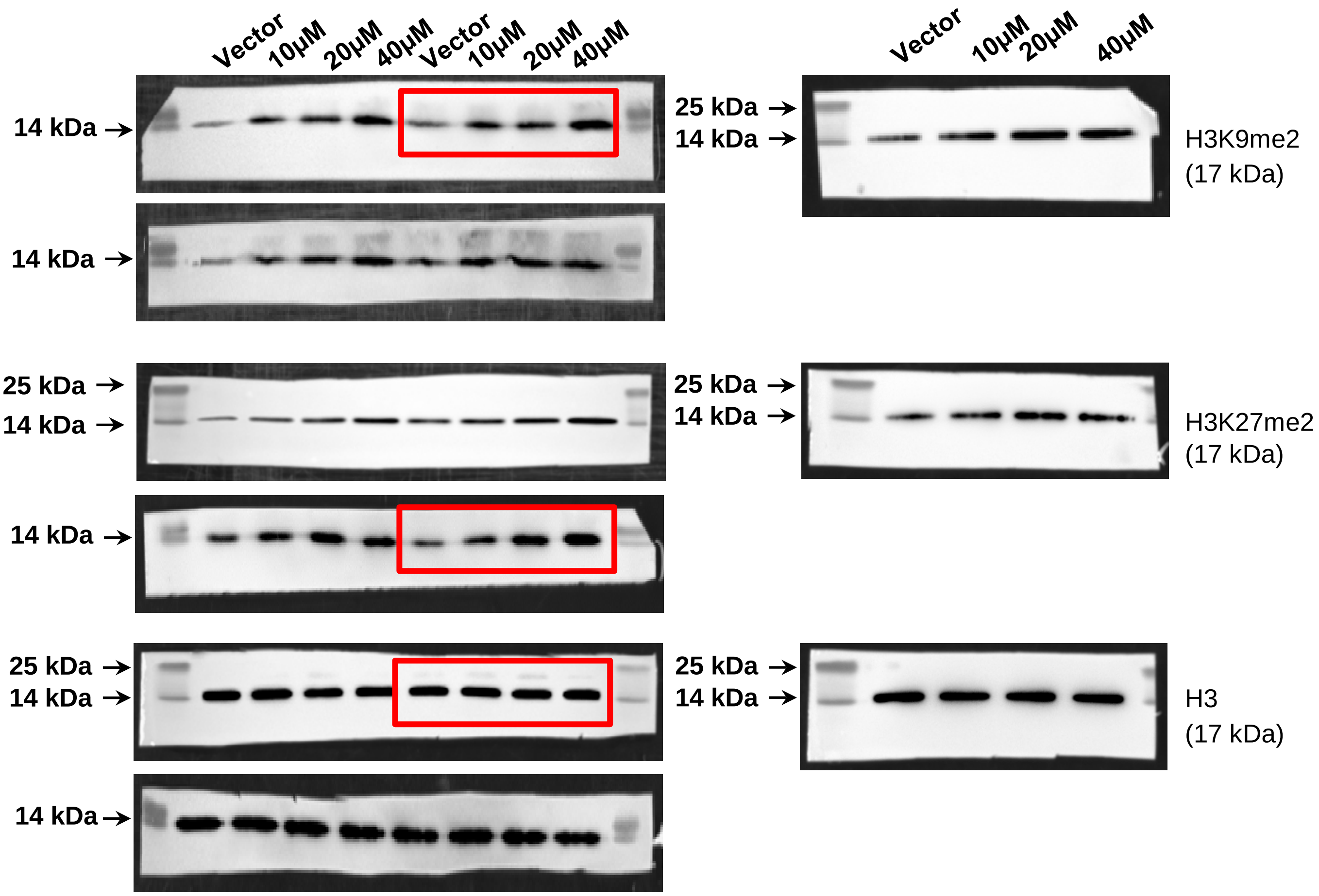

Vector
10μM
20μM
40μM
Vector
10μM
20μM
40μM
14 kDa
14 kDa
25 kDa
14 kDa
14 kDa
25 kDa
14 kDa
14 kDa
Vector
10μM
20μM
40μM
25 kDa
14 kDa
H3K9me2
(17 kDa)
25 kDa
14 kDa
H3K27me2
(17 kDa)
25 kDa
14 kDa
H3
(17 kDa)

Figure S6D

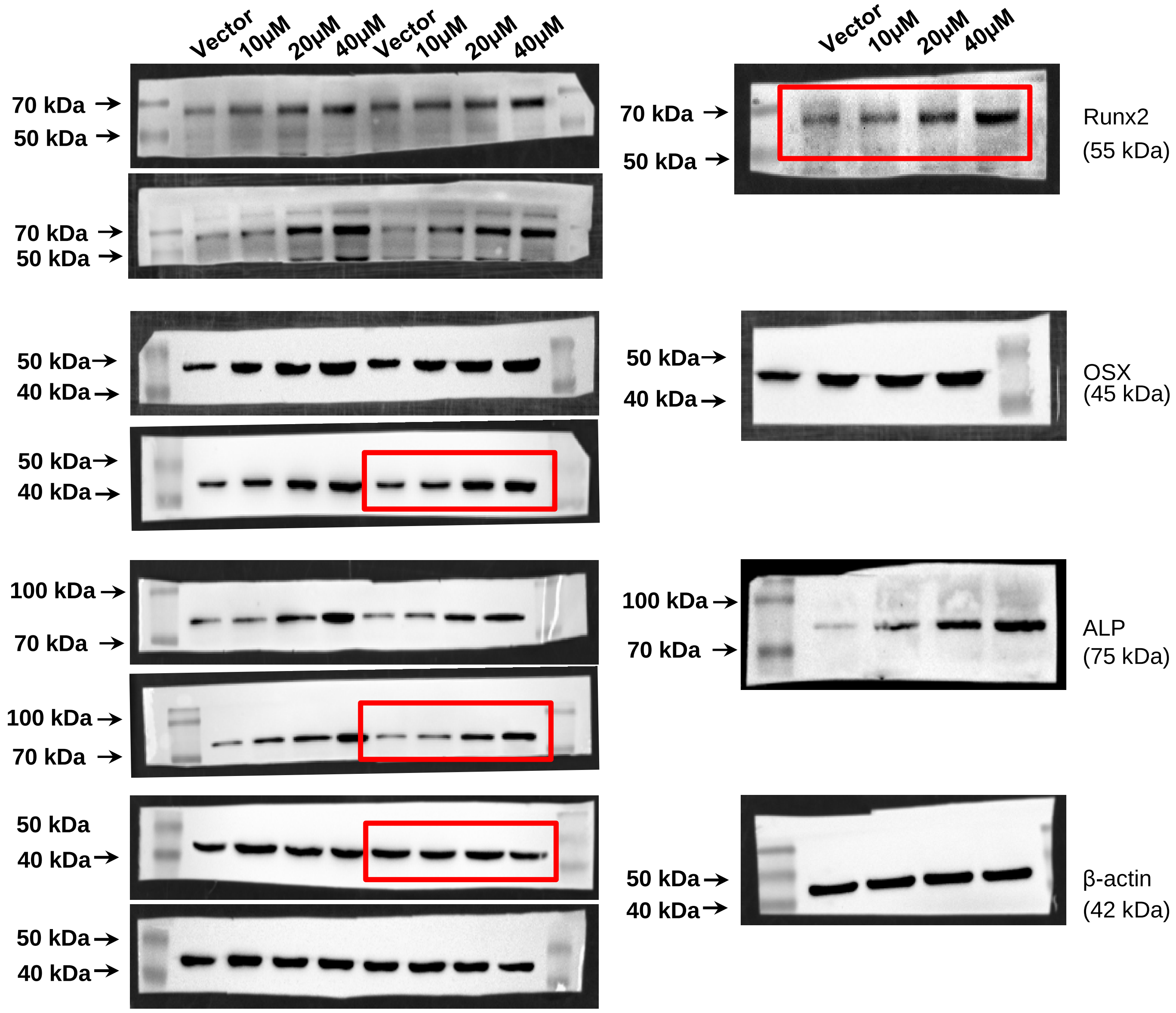
Vector
10μM
20μM
40μM
Vector
10μM
20μM
40μM
Vector
10μM
20μM
40μM
70 kDa
50 kDa
70 kDa
50 kDa
70 kDa
50 kDa
Runx2
(55 kDa)
50 kDa
40 kDa
50 kDa
40 kDa
50 kDa
40 kDa
OSX
(45 kDa)
100 kDa
70 kDa
100 kDa
70 kDa
100 kDa
70 kDa
ALP
(75 kDa)
50 kDa
40 kDa
50 kDa
40 kDa
50 kDa
40 kDa
β-actin
(42 kDa)

Figure S6H

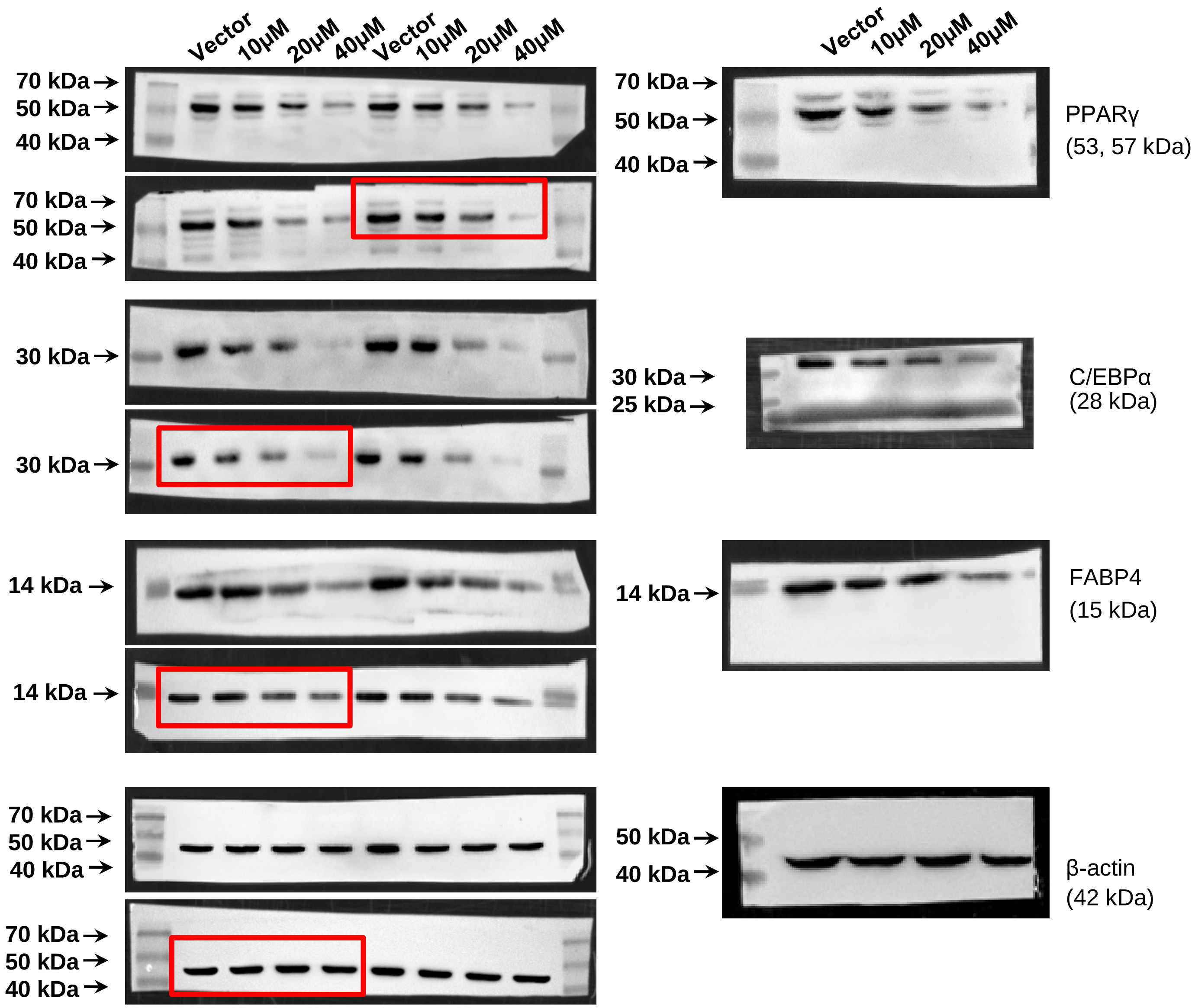

Vector
10μM
20μM
40μM
Vector
10μM
20μM
40μM
70 kDa
50 kDa
40 kDa
30 kDa
25 kDa
14 kDa
PPARγ
(53, 57 kDa)
C/EBPα
(28 kDa)
FABP4
(15 kDa)
β-actin
(42 kDa)

Figure S9D

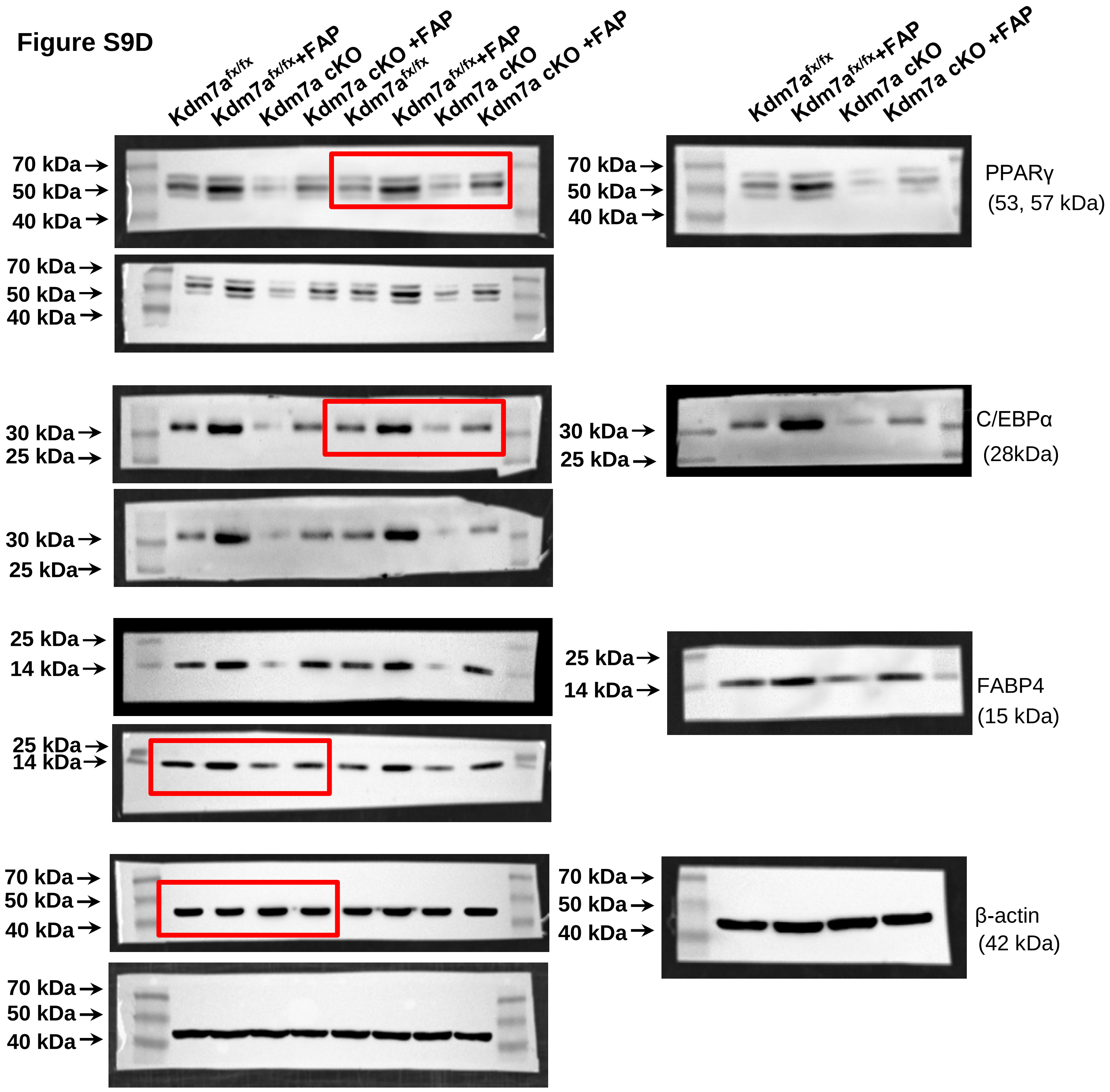
Kdm7a fx/fx
Kdm7a fx/fx +FAP
Kdm7a cKO
Kdm7a cKO +FAP
70 kDa
50 kDa
40 kDa
30 kDa
25 kDa
14 kDa
PPARγ
(53, 57 kDa)
C/EBPα
(28kDa)
FABP4
(15 kDa)
β-actin
(42 kDa)
