# Supplementary material for: Histone demethylase KDM7A regulates bone homeostasis through balancing osteoblast and osteoclast differentiation
Source: Cell Death Dis. 2024 Feb 12;15(2):136. doi: 10.1038/s41419-024-06521-z (PMC10861515; doi:10.1038/s41419-024-06521-z)
Supplement: Supplementary file 4 — Dataset 1 [file 41419_2024_6521_MOESM4_ESM.pdf]

RNA-Seq results

Gene ID	Gene Symbol	Type	log2 (Kdm7a cKO / Kdm7a$^{fx/fx}$)	FDR (Kdm7a cKO / Kdm7a$^{fx/fx}$)
11595	'Acan'	mRNA	-10.58871464	0.00E+00
11826	'Aqp1'	mRNA	-9.033423002	0.00E+00
12266	'C3'	mRNA	-8.271463028	0.00E+00
13179	'Dcn'	mRNA	-7.924812504	0.00E+00
13371	'Dio2'	mRNA	-7.826548487	0.00E+00
13516	'Epyc'	mRNA	-7.599912842	0.00E+00
14067	'F5'	mRNA	-5.754887502	0.00E+00
14219	'Ccn2'	mRNA	-5.554588852	0.00E+00
14560	'Gdf10'	mRNA	-4.196397213	0.00E+00
15439	'Hp'	mRNA	-3.67874853	0.00E+00
16181	'Il1rn'	mRNA	-3.321928095	0.00E+00
16819	'Lcn2'	mRNA	-3.253732223	0.00E+00
17022	'Lum'	mRNA	-3.209453366	0.00E+00
17386	'Mmp13'	mRNA	-3.185606624	0.00E+00
17392	'Mmp3'	mRNA	-3.169925001	0.00E+00
18619	'Penk'	mRNA	-3.054116549	0.00E+00
19241	'Tmsb4x'	mRNA	-3	0.00E+00
20257	'Stmn2'	mRNA	-2.981852653	0.00E+00
20657	'Sod3'	mRNA	-2.95419631	0.00E+00
21419	Tfap2b'	mRNA	-2.906890596	0.00E+00
216616	'Efemp1'	mRNA	-2.863263663	0.00E+00
21810	'Tgfbi'	mRNA	-2.856354323	0.00E+00
21826	'Thbs2'	mRNA	-2.813554181	0.00E+00
22003	'Tpm1'	mRNA	-2.784271309	0.00E+00
319480	'Itga11'	mRNA	-2.774657884	0.00E+00
50706	'Postn'	mRNA	-2.74607385	0.00E+00
78558	'Htra3'	mRNA	-2.736965594	0.00E+00
110454	'Ly6a'	mRNA	-2.678071905	3.75E-289
18162	Npr3'	mRNA	-2.667424661	1.29E-269
68588	'Cthrc1'	mRNA	-2.650253961	1.46E-263
74511	'Lrrc17'	mRNA	-2.62881679	3.08E-244
18126	'Nos2'	mRNA	-2.614709844	2.55E-243
12778	'Ackr3'	mRNA	-2.584962501	6.32E-227
17294	'Mest'	mRNA	-2.584962501	7.08E-226
380928	'Lmo7'	mRNA	-2.581422143	2.01E-206
12654	'Chil1'	mRNA	-2.580662363	2.01E-200
20515	Slc20a1'	mRNA	-2.572578776	6.36E-181

53867	Col5a3'	mRNA	-2.510961919	5.34E-180
12839	Col9a1'	mRNA	-2.4639471	5.36E-178
12841	'Col9a3'	mRNA	-2.458574148	5.07E-177
22418	Wnt5a'	mRNA	-2.432959407	2.04E-173
216285	'Alx1'	mRNA	-2.43171624	3.31E-168
26432	'Plod2'	mRNA	-2.415037499	3.46E-168
24059	Slco2a1'	mRNA	-2.403722186	2.47E-163
66222	Serpinb1a'	mRNA	-2.402098444	1.51E-159
23959	Nt5e'	mRNA	-2.396352936	1.09E-157
12159	'Bmp4'	mRNA	-2.383059455	5.94E-157
26908	'Eif2s3y'	mRNA	-2.377037731	4.32E-151
14836	'Gsc'	mRNA	-2.36923381	7.49E-146
16190	'Il4ra'	mRNA	-2.353636955	3.01E-144
23892	Grem1'	mRNA	-2.349584438	1.42E-141
12577	Cdkn1c'	mRNA	-2.331843564	1.91E-139
13835	Epha1'	mRNA	-2.286881148	4.96E-134
16840	Cnmd'	mRNA	-2.273018494	1.08E-133
26900	'Ddx3y'	mRNA	-2.250961574	3.12E-132
68655	Fndc1'	mRNA	-2.24469277	6.83E-128
320078	Olfml2b'	mRNA	-2.235628248	1.12E-126
16012	'Igfbp6'	mRNA	-2.219604604	4.86E-124
12022	Barx1'	mRNA	-2.207044457	1.46E-122
23937	Mab21l2'	mRNA	-2.182203331	2.48E-121
14461	Gata2'	mRNA	-2.169925001	1.47E-119
170768	Pfkfb3'	mRNA	-2.157676456	2.12E-117
15117	Has2'	mRNA	-2.133991014	4.00E-115
20502	Slc16a2'	mRNA	-2.132495172	3.35E-112
11535	Adm'	mRNA	-2.115477217	8.30E-112
21822	Tgtp1'	mRNA	-2.112089813	1.20E-110
19737	Rgs5'	mRNA	-2.094973598	2.04E-107
21943	Tnfsf11'	mRNA	-2.086826672	3.92E-107
16371	Irx1'	mRNA	-2.08451656	1.34E-106
19659	Rbp1'	mRNA	-2.035824933	9.51E-104
18511	Pax9'	mRNA	-2.034316059	3.60E-103
14758	Gpm6b'	mRNA	-2.02045061	1.12E-102
13876	Erg'	mRNA	-2.014260693	1.23E-100
17268	Meis1'	mRNA	-2.011227255	4.61E-100
64074	Smoc2'	mRNA	-2.009803175	5.23E-98
242553	Kank4'	mRNA	-2	4.30E-96
20723	Serpinb9'	mRNA	-1.991962681	2.25E-95

18491	Pappa'	mRNA	-1.978451798	4.03E-94
18111	Nnat'	mRNA	-1.928446739	7.51E-94
12642	Ch25h'	mRNA	-1.920565533	2.11E-92
18552	Pcsk5'	mRNA	-1.920565533	1.85E-91
72780	Rspo3'	mRNA	-1.906890596	5.21E-91
15122	'Hba-a1'	mRNA	-1.901819606	2.77E-90
18505	Pax3'	mRNA	-1.898732977	1.39E-88
64075	Smoc1'	mRNA	-1.894290559	4.52E-85
18549	'Pcsk2'	mRNA	-1.882643049	3.74E-81
11668	'Aldh1a1'	mRNA	-1.871675903	3.98E-79
26358	'Aldh1a7'	mRNA	-1.869192764	2.00E-76
12815	'Col11a2'	mRNA	-1.868255746	2.17E-76
20310	Cxcl2'	mRNA	-1.866084379	3.03E-75
12160	Bmp5'	mRNA	-1.859822342	1.00E-74
12810	Coch'	mRNA	-1.857980995	6.31E-74
18606	Enpp2'	mRNA	-1.856790358	9.27E-73
67784	Plxnd1'	mRNA	-1.844049722	1.27E-72
20708	Serpinb6b'	mRNA	-1.840521786	2.36E-72
18993	Pou3f3'	mRNA	-1.837025789	6.96E-72
59012	Moxd1'	mRNA	-1.836501268	1.41E-71
14049	Eya2'	mRNA	-1.832890014	2.79E-71
20592	'Kdm5d'	mRNA	-1.830074999	1.47E-70
17116	Mab21l1'	mRNA	-1.821029859	4.22E-70
68861	Dipk2a'	mRNA	-1.820611019	1.03E-66
20893	Bhlhe40'	mRNA	-1.818161677	4.76E-66
14432	Gap43'	mRNA	-1.817135943	1.63E-65
69540	Klk10'	mRNA	-1.810814624	8.13E-65
60531	Npvf'	mRNA	-1.790076931	1.11E-64
19283	Ptprz1'	mRNA	-1.789557976	5.83E-63
15229	Foxd1'	mRNA	-1.78506696	1.81E-62
96875	Prg4'	mRNA	-1.778951746	2.70E-62
54635	Pdgfc'	mRNA	-1.770518154	2.83E-62
18811	Prl2c2'	mRNA	-1.754722665	8.92E-62
17380	Mme'	mRNA	-1.750021747	2.07E-61
14261	Fmo1'	mRNA	-1.745070644	4.95E-61
14681	Gnao1'	mRNA	-1.743703254	2.81E-59
239250	Slitrk6'	mRNA	-1.742503778	2.85E-59
12950	Hapln1'	mRNA	-1.728009118	1.19E-58
20618	Sncg'	mRNA	-1.725936128	3.77E-57
94185	Tnfrsf21'	mRNA	-1.717600269	6.43E-57

17067	Ly6c1'	mRNA	-1.709290636	8.48E-57
215798	Adgrg6'	mRNA	-1.708537186	1.03E-55
330122	Cxcl3'	mRNA	-1.685848545	2.50E-55
21828	Thbs4'	mRNA	-1.67851384	1.33E-54
16668	Krt18'	mRNA	-1.678071905	1.65E-54
215653	Rassf2'	mRNA	-1.676958285	3.95E-54
12904	Crabp2'	mRNA	-1.673771768	1.08E-53
407790	Ndufa4l2'	mRNA	-1.670692375	1.48E-53
102637507	'Gm34298'	mRNA	-1.664660745	6.30E-53
110257	Hba-a2'	mRNA	-1.652076697	6.78E-53
22290	Uty'	mRNA	-1.647612151	3.75E-52
72361	Ces2g'	mRNA	-1.64689025	5.30E-51
107587	Osr2'	mRNA	-1.623308812	6.02E-51
16164	Il13ra1'	mRNA	-1.612976877	4.63E-50
12819	'Col15a1'	mRNA	-1.612183969	2.00E-49
12583	Cdo1'	mRNA	-1.607417352	5.61E-49
67547	Slc39a8'	mRNA	-1.602407635	6.43E-49
15216	Hfe'	mRNA	-1.599769977	1.14E-48
81799	C1qtnf3'	mRNA	-1.582771619	5.44E-48
77569	Limch1'	mRNA	-1.55818317	6.89E-48
217066	Elobl'	mRNA	-1.556591643	1.29E-47
17470	Cd200'	mRNA	-1.553935605	5.25E-47
11830	Aqp5'	mRNA	-1.552972386	7.98E-47
24117	Wif1'	mRNA	-1.552858236	1.42E-46
16691	Krt8'	mRNA	-1.551015169	3.63E-46
226075	Glis3'	mRNA	-1.54689446	4.87E-46
13628	Eef1a2'	mRNA	-1.546488353	1.45E-45
30060	Meltf'	mRNA	-1.543314803	1.80E-44
234356	Csgalnact1'	mRNA	-1.540568381	2.49E-44
16156	Il11'	mRNA	-1.540568381	9.78E-44
18828	Plscr2'	mRNA	-1.5334322	3.94E-43
15945	Cxcl10'	mRNA	-1.533123569	3.21E-42
11695	Alx4'	mRNA	-1.532728535	3.37E-42
17921	Myo7a'	mRNA	-1.530883867	8.94E-42
18605	Enpp1'	mRNA	-1.525583954	1.96E-41
114249	Npnt'	mRNA	-1.498637701	2.92E-41
216892	Spns2'	mRNA	-1.493188307	1.27E-40
18053	Ngfr'	mRNA	-1.487545956	1.06E-39
67451	Pkp2'	mRNA	-1.483082887	1.17E-39
215446	Entpd3'	mRNA	-1.481462387	1.60E-39

667742	Piezo2'	mRNA	-1.480066048	2.75E-39
11550	Adra1d'	mRNA	-1.47883415	7.53E-39
14013	Mecom'	mRNA	-1.47447914	2.24E-38
14563	Gdf5'	mRNA	-1.46712601	7.61E-38
93960	Nkd1'	mRNA	-1.465663572	7.88E-38
329251	Ppp1r12b'	mRNA	-1.459431619	9.23E-38
110310	Krt7'	mRNA	-1.451988635	9.99E-38
29818	Hspb7'	mRNA	-1.450252192	1.06E-37
12578	Cdkn2a'	mRNA	-1.436099115	2.12E-36
17182	'Matn3'	mRNA	-1.426897654	1.61E-34
12722	'Clca3a1'	mRNA	-1.41740156	4.62E-34
80877	'Lrba'	mRNA	-1.412693559	5.48E-34
19876	Robo1'	mRNA	-1.409938345	5.51E-34
268451	Rab11fip4'	mRNA	-1.408805546	1.10E-32
83925	Trps1'	mRNA	-1.408535933	2.43E-32
13824	'Epb41l4a'	mRNA	-1.402964667	3.06E-32
13717	Eln'	mRNA	-1.399747628	3.32E-32
228846	'D630003M21Rik'	mRNA	-1.399607459	7.12E-30
20665	Sox10'	mRNA	-1.398549376	8.93E-30
13798	En1'	mRNA	-1.397876948	1.30E-29
213119	Itga10'	mRNA	-1.394278939	1.69E-29
109700	Itga1'	mRNA	-1.392317423	3.94E-29
24056	Sh3bp5'	mRNA	-1.389652181	5.79E-29
14585	Gfra1'	mRNA	-1.389042291	2.52E-28
100039796	Tgtp2'	mRNA	-1.373458396	3.20E-28
242316	Gdf6'	mRNA	-1.370465872	2.28E-27
237979	Sdk2'	mRNA	-1.36923381	3.85E-27
12268	C4b'	mRNA	-1.366071622	1.34E-26
16669	Krt19'	mRNA	-1.363404731	7.81E-26
100689	Spon2'	mRNA	-1.3594028	7.99E-26
11474	Actn3'	mRNA	-1.353636955	9.88E-26
12840	Col9a2'	mRNA	-1.351472371	1.03E-25
11694	Alx3'	mRNA	-1.34692807	1.10E-25
263803	Pkn3'	mRNA	-1.344445531	1.91E-25
15234	Hgf'	mRNA	-1.341036918	5.91E-25
228662	Btbd3'	mRNA	-1.336895054	7.60E-25
64899	Lpin3'	mRNA	-1.336182079	1.11E-24
58802	Kcnmb4'	mRNA	-1.333126301	1.14E-24
14089	Fap'	mRNA	-1.33268879	1.58E-24
15891	Ibsp'	mRNA	-1.330957061	8.97E-24

11501	Adam8'	mRNA	-1.329752311	1.30E-23
11600	Angpt1'	mRNA	-1.327396308	1.53E-23
67606	Fibin'	mRNA	-1.325133761	2.98E-23
213391	Rassf4'	mRNA	-1.313890776	3.63E-23
94249	Slc24a3'	mRNA	-1.305626283	5.46E-23
17001	Ltc4s'	mRNA	-1.304854582	1.32E-22
77590	Chst15'	mRNA	-1.30256277	1.48E-22
20297	Ccl20'	mRNA	-1.299560282	1.89E-22
54156	Egfl6'	mRNA	-1.298081353	2.29E-22
94216	'Col4a6'	mRNA	-1.296182682	3.32E-22
59036	Dact1'	mRNA	-1.293953467	4.70E-22
73340	Nptxr'	mRNA	-1.289506617	6.98E-22
15228	Foxg1'	mRNA	-1.28757659	8.94E-22
19220	Ptgfr'	mRNA	-1.286118019	2.32E-21
23948	Mmp17'	mRNA	-1.285128177	3.67E-21
100503605	'Hbb-bs'	mRNA	-1.278301162	8.89E-21
20706	Serpinb9b'	mRNA	-1.275893617	1.26E-20
80910	Gpr84'	mRNA	-1.273719002	3.56E-20
330723	Htra4'	mRNA	-1.271804615	3.60E-20
18741	Pitx2'	mRNA	-1.267695783	9.45E-20
56224	Tspan5'	mRNA	-1.267480311	1.61E-19
16918	Mycl'	mRNA	-1.267068138	2.11E-19
56847	Aldh1a3'	mRNA	-1.264499815	3.34E-19
19206	Ptch1'	mRNA	-1.264415636	3.62E-19
269152	Kif26b'	mRNA	-1.263541593	4.54E-19
13602	Sparcl1'	mRNA	-1.263034406	5.57E-19
16170	Il16'	mRNA	-1.262349203	9.89E-19
11601	Angpt2'	mRNA	-1.262000586	1.33E-18
13617	Ednra'	mRNA	-1.261441148	1.93E-18
227358	Erfe'	mRNA	-1.259781428	4.08E-18
12985	Csf3'	mRNA	-1.25885873	4.59E-18
13809	Enpep'	mRNA	-1.257157839	4.87E-18
21386	'Tbx3'	mRNA	-1.245112498	6.68E-18
19662	Rbp4'	mRNA	-1.229246092	7.22E-18
11876	Artn'	mRNA	-1.228624375	7.42E-18
271970	Arsj'	mRNA	-1.227535725	1.00E-17
13390	Dlx1'	mRNA	-1.227068909	5.02E-17
78923	Chsy3'	mRNA	-1.224510139	7.70E-17
72634	Tdrkh'	mRNA	-1.222392421	2.87E-16
226866	Sbspon'	mRNA	-1.218384933	2.94E-16

56375	B4galt4'	mRNA	-1.211838643	3.03E-16
65221	Slc15a3'	mRNA	-1.206450877	4.26E-16
19016	Pparg'	mRNA	-1.205997128	4.47E-16
243958	Siglecg'	mRNA	-1.202714937	5.04E-16
14168	Fgf13'	mRNA	-1.201802017	6.07E-16
13123	Cyp7b1'	mRNA	-1.200119574	9.75E-16
110075	'Bmp3'	mRNA	-1.196397213	9.97E-16
18576	Pde3b'	mRNA	-1.189602486	1.16E-15
18740	Pitx1'	mRNA	-1.186401937	1.26E-15
14538	Gcnt2'	mRNA	-1.179259727	1.67E-15
83691	Crispld1'	mRNA	-1.178052882	2.10E-15
19229	Ptk2b'	mRNA	-1.175258446	2.24E-15
13032	Ctsc'	mRNA	-1.173064709	2.40E-15
14942	Gzme'	mRNA	-1.172767751	2.78E-15
104816	Aspg'	mRNA	-1.167944637	4.76E-15
219140	Spata13'	mRNA	-1.163285804	1.05E-14
237759	'Col23a1'	mRNA	-1.154187341	1.15E-14
54419	'Cldn6'	mRNA	-1.153623189	1.24E-14
70417	'Megf10'	mRNA	-1.150242636	3.43E-14
11770	Fabp4'	mRNA	-1.146005124	4.63E-14
14254	Flt1'	mRNA	-1.142957954	4.83E-14
18213	Ntrk3'	mRNA	-1.141355849	7.94E-14
14371	Fzd9'	mRNA	-1.140257886	1.58E-13
11551	Adra2a'	mRNA	-1.138784591	1.68E-13
240892	Dusp27'	mRNA	-1.137503524	2.00E-13
13396	Dlx6'	mRNA	-1.132351036	3.31E-13
68527	'Ucma'	mRNA	-1.12822571	3.63E-13
53321	Cntnap1'	mRNA	-1.12563311	4.10E-13
14007	Celf2'	mRNA	-1.121306296	5.58E-13
67020	Tmem88'	mRNA	-1.118458596	5.92E-13
70415	Stk26'	mRNA	-1.117491637	6.39E-13
108169105	'LOC108169105'	mRNA	-1.116659851	7.83E-13
21892	Tll1'	mRNA	-1.112894056	8.61E-13
140580	Elmo1'	mRNA	-1.112474729	8.85E-13
373864	Col27a1'	mRNA	-1.108524457	9.96E-13
30956	Aass'	mRNA	-1.106041637	2.25E-12
13837	Epha3'	mRNA	-1.10496956	2.26E-12
106565	Dlk2'	mRNA	-1.098680033	5.67E-12
171170	Mbnl3'	mRNA	-1.097663259	5.82E-12
320873	Cdh10'	mRNA	-1.097253459	6.51E-12

219134	Shisa2'	mRNA	-1.094841434	6.71E-12
330790	Hapln4'	mRNA	-1.092393604	6.81E-12
67664	Rnf125'	mRNA	-1.091147888	7.27E-12
239618	Pdzrn4'	mRNA	-1.088024748	8.02E-12
12020	Nkx3-2'	mRNA	-1.087785484	8.91E-12
320916	Wscd2'	mRNA	-1.086622355	1.30E-11
16565	Kif21b'	mRNA	-1.085079243	1.62E-11
171463	Il17rd'	mRNA	-1.084474568	2.01E-11
228564	Frmd5'	mRNA	-1.081427599	2.18E-11
67621	Bend5'	mRNA	-1.080401507	2.18E-11
20666	Sox11'	mRNA	-1.078002512	2.32E-11
56198	Heyl'	mRNA	-1.073229911	2.56E-11
218772	Rarb'	mRNA	-1.072669068	3.41E-11
226041	Pgm5'	mRNA	-1.072485507	3.44E-11
544963	Iqgap2'	mRNA	-1.069708972	3.80E-11
215632	Psd4'	mRNA	-1.068809158	4.59E-11
68312	Gstm7'	mRNA	-1.066437985	4.81E-11
29815	Bcar3'	mRNA	-1.065802058	5.65E-11
268354	Tafa2'	mRNA	-1.06511729	7.01E-11
320365	Fry'	mRNA	-1.061927749	9.21E-11
103889	Hoxb2'	mRNA	-1.060598825	9.30E-11
107303348	'Gm45935'	mRNA	-1.058893689	9.71E-11
56047	Msln'	mRNA	-1.057600267	1.04E-10
20292	Ccl11'	mRNA	-1.055495113	1.31E-10
54525	Syt7'	mRNA	-1.054447784	1.33E-10
170706	Tmem37'	mRNA	-1.054447784	1.65E-10
11459	Acta1'	mRNA	-1.052396836	2.00E-10
20556	Slfn2'	mRNA	-1.050561547	2.08E-10
210719	Mkx'	mRNA	-1.049468676	2.55E-10
14675	Gna14'	mRNA	-1.048738655	2.70E-10
70967	Eva1c'	mRNA	-1.045514659	4.17E-10
333564	Fndc3c1'	mRNA	-1.042310805	4.32E-10
320865	Cdh18'	mRNA	-1.03932313	4.64E-10
170677	Cdhr1'	mRNA	-1.038243835	5.01E-10
74199	Vit'	mRNA	-1.037474705	5.37E-10
94227	Pi15'	mRNA	-1.036525876	7.01E-10
225608	Sh3tc2'	mRNA	-1.034036324	9.33E-10
243659	Styk1'	mRNA	-1.033964898	9.61E-10
16416	Itgb3'	mRNA	-1.029747343	9.63E-10
19049	Ppp1r1b'	mRNA	-1.029210817	1.26E-09

233781	Xylt1'	mRNA	-1.028569152	1.65E-09
241494	Zfp385b'	mRNA	-1.026472211	1.98E-09
71145	Scara5'	mRNA	-1.025928465	2.11E-09
118449	Synpo2'	mRNA	-1.024662054	4.83E-09
13592	Ebf2'	mRNA	-1.021061616	5.29E-09
237523	Ptprq'	mRNA	-1.018650057	5.99E-09
330406	B4galnt3'	mRNA	-1.015266757	6.08E-09
12182	Bst1'	mRNA	-1.013674937	7.00E-09
20503	Slc16a7'	mRNA	-1.01199155	7.37E-09
214112	Nipal4'	mRNA	-1.003598643	8.70E-09
18417	Cldn11'	mRNA	-1.002601795	9.21E-09
218215	Rnf144b'	mRNA	-1	1.10E-08
14176	'Fgf5'	mRNA	1	1.11E-08
17342	'Mitf'	mRNA	1	1.15E-08
69908	'Rab3b'	mRNA	1.004898799	1.40E-08
57738	'Slc15a2'	mRNA	1.008988783	1.42E-08
67874	'Rprm'	mRNA	1.009715155	1.47E-08
110557	'H2-Q6'	mRNA	1.012600037	1.57E-08
242939	'Cpz'	mRNA	1.016119665	1.90E-08
50759	'Fbxo16'	mRNA	1.016873819	1.91E-08
11670	'Aldh3a1'	mRNA	1.01720929	2.19E-08
13867	'Erbb3'	mRNA	1.021216486	3.32E-08
17968	'Ncam2'	mRNA	1.030422921	3.42E-08
99526	'Usp53'	mRNA	1.033683126	3.75E-08
18158	'Nppb'	mRNA	1.036174788	4.02E-08
224090	'Tmem44'	mRNA	1.041211117	4.43E-08
14017	'Evi2a'	mRNA	1.046459311	5.10E-08
18143	'Npas2'	mRNA	1.048661582	5.25E-08
16174	'Il18rap'	mRNA	1.050040682	5.28E-08
101488143	'Hbb-bt'	mRNA	1.052974465	5.39E-08
12740	'Cldn4'	mRNA	1.054447784	5.39E-08
240753	'Plekha6'	mRNA	1.060925704	6.22E-08
269642	'Nat8l'	mRNA	1.068838876	6.41E-08
66607	'Ms4a4d'	mRNA	1.068838876	6.56E-08
246228	'Vwa1'	mRNA	1.08246216	6.92E-08
16782	'Lamc2'	mRNA	1.088024748	7.34E-08
234673	'Ces2e'	mRNA	1.096676019	7.78E-08
327814	'Ppfia2'	mRNA	1.096945556	7.95E-08
71690	'Esm1'	mRNA	1.100868828	1.05E-07
74342	'Lrrtm1'	mRNA	1.104835209	1.07E-07

243743	'Plxna4'	mRNA	1.108209217	1.34E-07
100043381	'Gm14308'	mRNA	1.114536432	1.56E-07
170799	'Rtkn2'	mRNA	1.12241544	1.56E-07
57776	'Ttyh1'	mRNA	1.12268252	1.58E-07
271127	'Adamts16'	mRNA	1.136866713	1.59E-07
81877	'Tnxb'	mRNA	1.141661149	2.23E-07
11630	'Crybg1'	mRNA	1.145850866	2.54E-07
627626	'Ptchd4'	mRNA	1.156725504	2.61E-07
14462	'Gata3'	mRNA	1.158989524	2.86E-07
269295	'Rtn4rl2'	mRNA	1.160776325	2.98E-07
17395	'Mmp9'	mRNA	1.162271429	2.98E-07
18772	'Pkp1'	mRNA	1.182591413	3.08E-07
11302	'Aatk'	mRNA	1.185555653	3.31E-07
20776	'Tmie'	mRNA	1.187725289	3.34E-07
15371	'Hmx1'	mRNA	1.190331212	4.19E-07
14579	'Gem'	mRNA	1.200593831	4.89E-07
12323	'Camk2b'	mRNA	1.201633861	4.89E-07
20128	'Trim30a'	mRNA	1.215142682	5.06E-07
76487	'Ppp1r3g'	mRNA	1.217962897	5.09E-07
245020	'Slc35g2'	mRNA	1.222392421	6.31E-07
319171	'H2ac24'	mRNA	1.228203261	7.20E-07
73167	'Arhgap8'	mRNA	1.240051088	7.26E-07
15410	'Hoxb3'	mRNA	1.242856524	7.66E-07
68553	'Col6a4'	mRNA	1.270701259	8.87E-07
434341	'Nlrc5'	mRNA	1.284177324	9.13E-07
71889	'Epn3'	mRNA	1.289891343	9.38E-07
12404	'Cbln1'	mRNA	1.291933355	9.56E-07
320840	'Negr1'	mRNA	1.29225274	9.59E-07
16494	'Kcna6'	mRNA	1.300910037	1.05E-06
54448	'Il1f6'	mRNA	1.311944006	1.09E-06
15077	'H3c14'	mRNA	1.328836464	1.09E-06
17286	'Meox2'	mRNA	1.336716591	1.11E-06
229898	'Gbp5'	mRNA	1.33848275	1.32E-06
54140	'Avpr1a'	mRNA	1.356338126	1.53E-06
232035	'Ccser1'	mRNA	1.370223652	1.72E-06
70638	'Fam189a1'	mRNA	1.381870635	1.74E-06
16858	'Lgals7'	mRNA	1.38310701	1.77E-06
22422	'Wnt7b'	mRNA	1.391128356	2.09E-06
115490184	'Gm42427'	mRNA	1.393481356	2.11E-06
80797	'Clca3a2'	mRNA	1.394859617	2.28E-06

11567	'Avil'	mRNA	1.395009019	2.43E-06
73121	'Rflna'	mRNA	1.396517411	2.69E-06
23859	'Dlg2'	mRNA	1.398549376	2.94E-06
12061	'Bdkrb1'	mRNA	1.406940032	3.00E-06
78921	'9130019O22Rik'	mRNA	1.408250015	3.38E-06
19017	'Ppargc1a'	mRNA	1.411015811	3.50E-06
15412	'Hoxb4'	mRNA	1.422233001	3.58E-06
12224	'Klf5'	mRNA	1.429880836	3.69E-06
22774	'Zic4'	mRNA	1.449610648	3.71E-06
208098	'Panx3'	mRNA	1.459112474	4.11E-06
58237	'Nkain4'	mRNA	1.461843481	4.45E-06
231440	'Parm1'	mRNA	1.465663572	4.56E-06
433022	'Plcxd2'	mRNA	1.478653599	4.63E-06
66042	'Sostdc1'	mRNA	1.505109193	4.81E-06
207742	'Rnf43'	mRNA	1.510961919	4.95E-06
24084	'Tekt2'	mRNA	1.514101627	5.37E-06
56089	'Ramp3'	mRNA	1.518928017	5.39E-06
12349	'Car2'	mRNA	1.526545814	5.89E-06
106952	'Arap3'	mRNA	1.528100164	6.30E-06
59058	'Bhlhe22'	mRNA	1.528198681	6.48E-06
213522	'Plekhg6'	mRNA	1.559342382	6.69E-06
16875	'Lhx8'	mRNA	1.567040593	7.20E-06
16196	'Il7'	mRNA	1.568842835	7.20E-06
17311	'Kitl'	mRNA	1.572989859	7.39E-06
76469	'Cmya5'	mRNA	1.584962501	7.72E-06
11847	'Arg2'	mRNA	1.584962501	7.72E-06
271424	'Ip6k3'	mRNA	1.598078001	7.72E-06
11307	'Abcg1'	mRNA	1.605100146	8.39E-06
24131	'Ldb3'	mRNA	1.610053482	8.83E-06
15245	'Hhip'	mRNA	1.616821415	9.85E-06
78748	'Rassf10'	mRNA	1.662071426	1.02E-05
17231	'Mcpt8'	mRNA	1.674599713	1.10E-05
115489690	'Gm52606'	mRNA	1.691755891	1.11E-05
16538	'Kcns1'	mRNA	1.726981506	1.14E-05
14960	'H2-Aa'	mRNA	1.732171445	1.23E-05
230822	'Ncmap'	mRNA	1.734619575	1.34E-05
270097	'Vat1l'	mRNA	1.736965594	1.51E-05
57329	'Otor'	mRNA	1.776731504	1.57E-05
226695	'Ifi205'	mRNA	1.777607579	1.68E-05
16456	'F11r'	mRNA	1.777844222	1.79E-05

21682	'Tec'	mRNA	1.786596362	1.81E-05
59095	'Fxyd6'	mRNA	1.789320999	1.81E-05
231162	'Cytl1'	mRNA	1.807354922	1.98E-05
108167434	'Gm45988'	mRNA	1.819427754	2.02E-05
93735	'Wnt16'	mRNA	1.821662759	2.06E-05
241226	'Itga8'	mRNA	1.831877241	2.19E-05
239650	'Ccdc184'	mRNA	1.833375167	2.34E-05
140795	'P2ry14'	mRNA	1.84502534	2.58E-05
12801	'Cnr1'	mRNA	1.857980995	2.62E-05
69253	'Hspb2'	mRNA	1.888919532	2.89E-05
97998	'Deptor'	mRNA	1.900239988	2.94E-05
93842	'Igsf9'	mRNA	1.905997512	3.05E-05
20358	'Sema6a'	mRNA	1.906890596	3.10E-05
115489125	'Gm52435'	mRNA	1.91346325	3.30E-05
16409	'Itgam'	mRNA	1.921997488	3.44E-05
16572	'Kif5a'	mRNA	1.94116897	3.72E-05
14190	'Fgl2'	mRNA	1.967938791	4.12E-05
622434	'Arhgef26'	mRNA	1.986060809	4.83E-05
12518	'Cd79a'	mRNA	1.99794926	5.08E-05
230726	'Rhbdl2'	mRNA	2	5.08E-05
68027	'Tmem178'	mRNA	2.016258585	6.14E-05
83453	'Chrdl1'	mRNA	2.018378529	6.47E-05
214305	'Hhipl1'	mRNA	2.026152288	7.20E-05
74306	'Prss46'	mRNA	2.030847588	8.09E-05
68339	'Ccdc88c'	mRNA	2.041820176	8.48E-05
56811	'Dkk2'	mRNA	2.041982449	8.62E-05
14941	'Gzmd'	mRNA	2.042644337	8.87E-05
18812	'Prl2c3'	mRNA	2.053111336	9.99E-05
20284	'Scrg1'	mRNA	2.0669787	1.04E-04
12391	'Cav3'	mRNA	2.079019999	1.06E-04
12737	'Cldn1'	mRNA	2.120294234	1.23E-04
59308	'Emcn'	mRNA	2.186998515	1.23E-04
268595	'D430019H16Rik'	mRNA	2.192223669	1.27E-04
74175	'Crct1'	mRNA	2.192645078	1.27E-04
12156	'Bmp2'	mRNA	2.222392421	1.29E-04
72027	'Slc39a4'	mRNA	2.286522759	1.29E-04
236573	'Gbp9'	mRNA	2.28757659	1.29E-04
319387	'Adgrl3'	mRNA	2.337409082	1.32E-04
234857	'Spire2'	mRNA	2.338662111	1.45E-04
58916	'Myot'	mRNA	2.347923303	1.61E-04

74901	'Kbtbd11'	mRNA	2.350497247	1.66E-04
20269	'Scn3a'	mRNA	2.407847121	1.68E-04
54418	'Fmn2'	mRNA	2.422847328	1.78E-04
21789	'Tfpi2'	mRNA	2.43718554	1.79E-04
319772	'C130050O18Rik'	mRNA	2.45169597	1.82E-04
53374	'Chst3'	mRNA	2.459431619	1.82E-04
22271	'Upp1'	mRNA	2.472068444	1.83E-04
13052	'Cxadr'	mRNA	2.523561956	1.86E-04
12739	'Cldn3'	mRNA	2.5360529	1.89E-04
50765	'Tfr2'	mRNA	2.563136138	1.94E-04
67425	'Eps8l1'	mRNA	2.576599028	2.05E-04
14465	'Gata6'	mRNA	2.584962501	2.13E-04
16644	'Kng1'	mRNA	2.624490865	2.14E-04
13118	'Cyp4a12b'	mRNA	2.64625868	2.19E-04
54710	'Hs3st3b1'	mRNA	2.652076697	2.20E-04
171209	'Asic3'	mRNA	2.724892762	2.24E-04
18132	'Notch4'	mRNA	2.73039294	2.41E-04
232441	'Rerg'	mRNA	2.746243408	2.51E-04
231655	'Oasl1'	mRNA	2.836050355	2.51E-04
192167	'Nlgn1'	mRNA	2.84556608	2.56E-04
235106	'Ntm'	mRNA	2.867452284	2.56E-04
14867	'Gstm6'	mRNA	2.906890596	2.95E-04
20259	'Scin'	mRNA	2.924759016	2.99E-04
622645	'Tmem200c'	mRNA	3	3.06E-04
192663	'Abcg4'	mRNA	3.106422059	3.10E-04
241035	'Pkhd1'	mRNA	3.169925001	3.59E-04
380713	'Scarf1'	mRNA	3.169925001	3.62E-04
634882	'Itih5l-ps'	mRNA	3.247927513	3.62E-04
109979	'Art3'	mRNA	3.274584514	3.62E-04
12167	'Bmpr1b'	mRNA	3.303055362	3.66E-04
233020	'Hipk4'	mRNA	3.350497247	3.80E-04
232714	'Mgam'	mRNA	3.356338126	3.84E-04
100039210	'Gm2102'	mRNA	3.374518922	3.84E-04
213573	'Cracr2b'	mRNA	3.375039431	3.85E-04
13024	'Ctla2a'	mRNA	3.419538892	3.96E-04
228413	'Prrg4'	mRNA	3.652076697	4.04E-04
407800	'Ecm2'	mRNA	3.709658248	4.14E-04
18509	'Pax7'	mRNA	3.733354341	4.39E-04
66610	'Abi3'	mRNA	3.781359714	4.39E-04
15402	'Hoxa5'	mRNA	3.805010982	4.52E-04

14160	'Lgr5'	mRNA	3.955389113	4.68E-04
23880	'Fyb'	mRNA	3.977279923	4.73E-04
100043920	'Fam205a4'	mRNA	3.994353437	5.79E-04
74134	'Cyp2s1'	mRNA	4	5.89E-04
233744	'Spon1'	mRNA	4	6.17E-04
50795	'Sh3bgr'	mRNA	4.075288127	6.21E-04
76088	'Dock8'	mRNA	4.115477217	6.28E-04
15375	'Foxa1'	mRNA	4.254565992	6.53E-04
330119	'Adamts3'	mRNA	4.310528633	6.55E-04
107146	'Glyat'	mRNA	4.311748315	6.71E-04
12971	'Crym'	mRNA	4.509013647	6.73E-04
554327	'2610042L04Rik'	mRNA	4.526286593	6.78E-04
15446	'Hpgd'	mRNA	4.906890596	6.78E-04
102640594	'Gm28710'	mRNA	5.077637411	7.94E-04
16511	'Kcnh2'	mRNA	5.106199404	7.99E-04
56222	'Cited4'	mRNA	5.426264755	8.27E-04
64706	'Scube1'	mRNA	5.523561956	8.32E-04
72125	'Amer2'	mRNA	5.608809243	8.71E-04
236576	'Spry3'	mRNA	5.844095469	8.74E-04
15478	'Hs3st3a1'	mRNA	5.857980995	9.33E-04
353169	'Slc2a12'	mRNA	6.285402219	9.36E-04
260315	'Nav3'	mRNA	7.22881869	9.39E-04
18190	'Nrxn2'	mRNA	8.018200179	9.78E-04
58185	'Rsad2'	mRNA	9.041659152	9.86E-04
